# Gut-first Parkinson’s disease is encoded by gut dysbiome

**DOI:** 10.1186/s13024-024-00766-0

**Published:** 2024-10-24

**Authors:** Mário F. Munoz-Pinto, Emanuel Candeias, Inês Melo-Marques, A. Raquel Esteves, Ana Maranha, João D. Magalhães, Diogo Reis Carneiro, Mariana Sant’Anna, A. Raquel Pereira-Santos, António E Abreu, Daniela Nunes-Costa, Susana Alarico, Igor Tiago, Ana Morgadinho, João Lemos, Pedro N. Figueiredo, Cristina Januário, Nuno Empadinhas, Sandra Morais Cardoso

**Affiliations:** 1 Center for Neuroscience and Cell Biology, University of Coimbra, Coimbra, Portugal; 2 Centre for Innovative Biomedicine and Biotechnology, University of Coimbra, Coimbra, Portugal; 3 PhD Programme in Experimental Biology and Biomedicine (PDBEB), Institute for Interdisciplinary Research, University of Coimbra, Coimbra, Portugal; 4 Department of Neurology, CHUC – Centro Hospitalar e Universitário de Coimbra, Coimbra, Portugal; 5 Faculty of Medicine, University of Coimbra, Coimbra, Portugal; 6 Department of Gastroenterogy, CHUC – Centro Hospitalar e Universitário de Coimbra, Coimbra, Portugal; 7 Centre for Functional Ecology, University of Coimbra, Coimbra, Portugal; 8 Present affiliation: Department of Biochemistry and Molecular Biology, Faculty of Pharmacy, University of Seville, Seville, Spain

**Keywords:** Parkinson’s disease, Gut microbiome, Dysbiosis, Dysbiome, Innate immunity, Gut-brain axis, Inflammation, Mitochondria, Dopaminergic, Dorsal motor nucleus of the vagus, Substantia nigra

## Abstract

**Background:**

In Parkinson's patients, intestinal dysbiosis can occur years before clinical diagnosis, implicating the gut and its microbiota in the disease. Recent evidence suggests the gut microbiota may trigger body**-**first Parkinson Disease (PD), yet the underlying mechanisms remain unclear. This study aims to elucidate how a dysbiotic microbiome through intestinal immune alterations triggers PD**-**related neurodegeneration.

**Methods:**

To determine the impact of gut dysbiosis on the development and progression of PD pathology, wild-type male C57BL/6 mice were transplanted with fecal material from PD patients and age**-**matched healthy donors to challenge the gut-immune-brain axis.

**Results:**

This study demonstrates that patient-derived intestinal microbiota caused midbrain tyrosine hydroxylase positive (TH +) cell loss and motor dysfunction. Ileum-associated microbiota remodeling correlates with a decrease in Th17 homeostatic cells. This event led to an increase in gut inflammation and intestinal barrier disruption. In this regard, we found a decrease in CD4 + cells and an increase in pro-inflammatory cytokines in the blood of PD transplanted mice that could contribute to an increase in the permeabilization of the blood–brain-barrier, observed by an increase in mesencephalic Ig**-**G**-**positive microvascular leaks and by an increase of mesencephalic IL**-**17 levels, compatible with systemic inflammation. Furthermore, alpha-synuclein aggregates can spread caudo-rostrally, causing fragmentation of neuronal mitochondria. This mitochondrial damage subsequently activates innate immune responses in neurons and triggers microglial activation.

**Conclusions:**

We propose that the dysbiotic gut microbiome (dysbiome) in PD can disrupt a healthy microbiome and Th17 homeostatic immunity in the ileum mucosa, leading to a cascade effect that propagates to the brain, ultimately contributing to PD pathophysiology. Our landmark study has successfully identified new peripheral biomarkers that could be used to develop highly effective strategies to prevent the progression of PD into the brain.

**Graphical Abstract:**

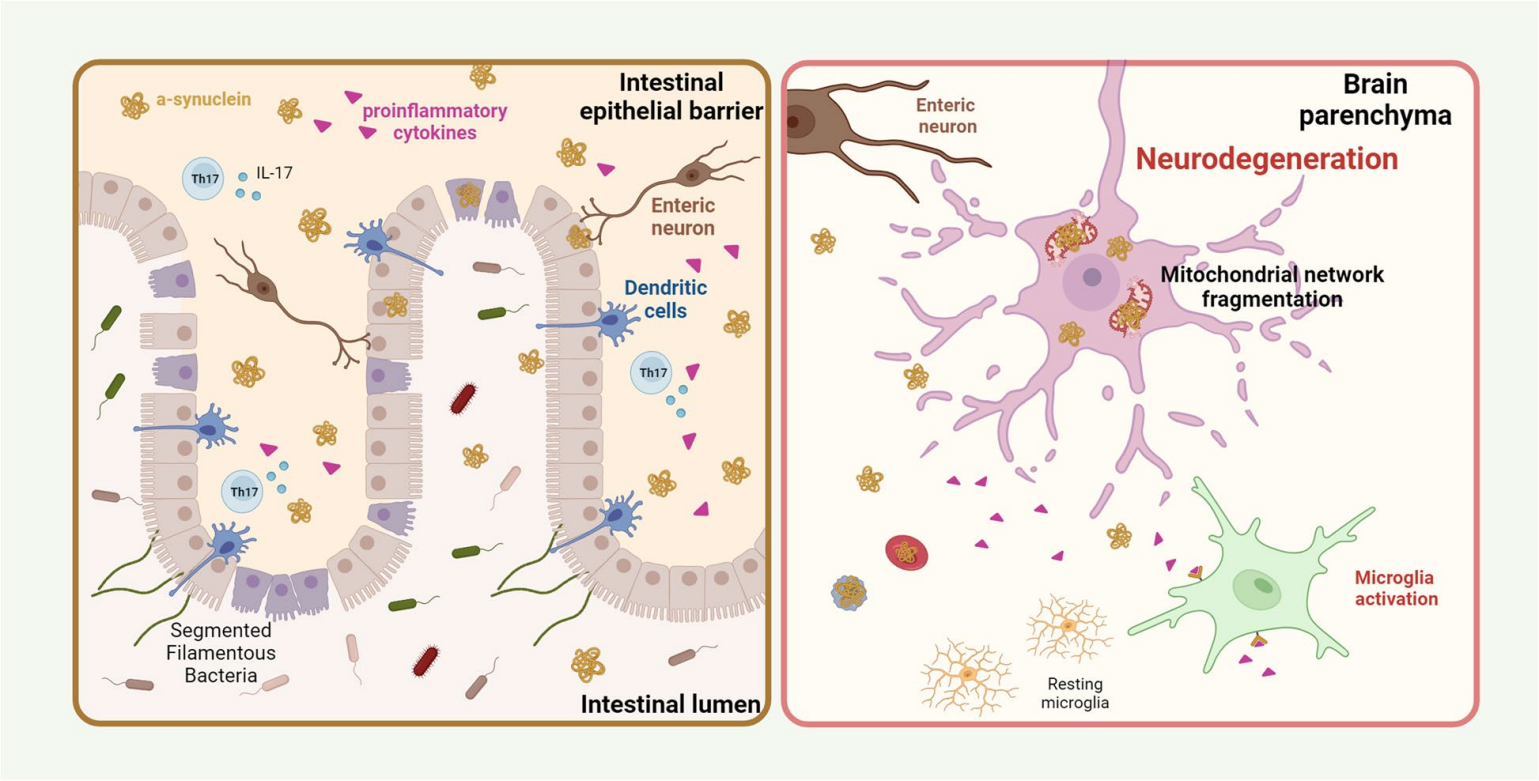

**Supplementary Information:**

The online version contains supplementary material available at 10.1186/s13024-024-00766-0.

## Background

The mammalian gut hosts intricate microbial communities engaged in constant interactions, employing both cooperative and competitive mechanisms to optimize resource utilization and coexistence [1]. While some microbes benefit from nutrients produced by their counterparts, others employ tactics such as releasing toxic metabolites or effectors to eliminate competitors. [1]. This delicate balance can be disrupted in cases of gut dysbiosis, increasingly linked with Parkinson’s disease (PD) [2, 3], leading to the proliferation of pathobionts and chronic production of toxins targeting vital bacterial commensals. Remarkably, this dysbiosis can extend its impact to mitochondria, including those within neurons [4]. Braak and colleagues proposed a hypothesis suggesting that the pathogenesis of PD could potentially initiate in the enteric nervous system (ENS), with potential access to the brain through the entire gastrointestinal (GI) tract [5]. This has led to the hypothesis that an exogenous toxin or pathogen can trigger and spread the disease via retrograde axonal vagus transport from the ENS to the Central Nervous System (CNS) [6]. It’s worth noting that gastrointestinal symptoms often precede motor manifestations in PD, underlining the involvement of the gut in disease onset [7, 8]. Furthermore, complete truncal vagotomy significantly reduced the risk of developing PD [9], indicating the crucial involvement of the vagus nerve in disease pathogenesis. The idea that pathogenic aSyn could potentially spread from the gut to the brain, leading to the degeneration of the nigrostriatal dopaminergic system, is indeed intriguing [10, 11]. Furthermore, the microbes can influence aSyn overexpression, as evidenced by different studies showing dopaminergic neuronal damage, neuroinflammation, and motor deficits in bacterial infected mice [12–14]. PD patients exhibit significantly different fecal and mucosa-associated microbiota compared to healthy controls [3, 15, 16], with PD patients experiencing exacerbated intestinal inflammation [17]. Dysbiosis in the gut has been proposed to induce monocyte and T-cell infiltration into the brain parenchyma [18, 19], driving both local brain inflammatory responses and engagement of peripheral immune mechanisms [20]. Intestinal inflammation is associated with the loss of tissue-resident homeostatic intestinal T helper (Th) 17 cells, crucial for protecting the gut barrier integrity [21, 22]. However, a dysregulated Th17 cell response can promote chronic inflammation, contributing to systemic inflammation and loss of blood–brain barrier (BBB) integrity [23].

Although individual genetic factors likely play a role in triggering gut dysbiosis leading to intestinal barrier loss, systemic low-grade chronic inflammation, and blood–brain barrier (BBB) leakage, recent findings indicate that patients with inflammatory bowel disease (IBD) face a significantly higher risk of developing PD [24–26].

The compromised BBB, essential for CNS homeostasis, is also observed in PD patients [27, 28]. Thus, the disruption of BBB and recruitment of peripheral immune cells seem pivotal in PD progression [18, 19]. However, the precise mechanisms by which PD gut microbiota induce intestinal barrier disruption or BBB dysfunction remains understood. While signatures of PD gut microbiota are emerging [29, 30], their functional implications remain largely elusive. Nevertheless, toxins from certain gut microbes have been shown to damage mitochondria, potentially contributing to PD onset [4, 31, 32]. Our mitochondrial cascade hypothesis for PD [33], suggests that dysfunctional mitochondria, possibly due to gut dysbiosis, induce neuronal innate immune activation, emphasizing microbiome-mitochondria crosstalk in PD neurodegeneration [34].

Studies indicate that fecal transplantation can alter host microbial communities and microbe-host interactions [35]. Transplantation from young to aged mice has shown beneficial effects on the gut-brain axis, while the reverse led to detrimental brain changes [36, 37]. Furthermore, colonization of PD genetic mice model with gut microbiota from PD patients accelerated PD-like symptoms compared to healthy donor transplants [12].

We propose that fecal material from PD patients disrupts microbiota-host immune homeostasis, inducing PD-like symptoms in WT mice. The fecal ecosystem of PD patients contributes to dopaminergic neuronal loss and motor deficits in mice by impacting the ileum mucosa-associated microbiota and altering gut immune cell responses, promoting a pro-inflammatory gut environment disrupting intestinal barrier integrity. Moreover, aSyn pathology observed in the gut progresses to the brain, in response to innate immunity activation, leading to neuroinflammation. Collectively, our findings suggest that the persistent presence of a dysbiotic microbiome (dysbiome) of PD patients is sufficient to trigger pathology.

## Methods

### Human donors

Fecal material from human donors was obtained from patients followed at the Movement Disorders Unit of the Centro Hospitalar e Universitário de Coimbra. Outpatients with PD were recruited from outpatient clinics based on the following inclusion criteria: (a) meet the diagnosis of idiopathic PD according to the UK Brain Bank diagnostic criteria [38], (b) disease onset > 50 years of age (c) to have symptoms suggestive of constipation. As exclusion criteria: (a) hepatic, renal or cardiac failure, (b) severe hypertension, (c) other neurological disease, (d) head trauma, (e) any anti-inflammatory, antineoplastic or immunosuppressive treatment in the 3 months prior to sampling, drugs treatments, (f) any antibiotic treatment on the 3 months before sampling. Inclusion criteria for control subjects were limited to age > 50 years, and exclusion criteria were the same plus (a) consanguinity to any of the recruited individuals diagnosed with PD (b) family history of PD.

Blood samples were collected in anticoagulant tubes during a clinical visit by a trained practitioner as described below.

Ileal biopsies were performed by gastroenterology experts. A small piece of the terminal ileum (1–3 mm in diameter) was removed with a biopsy needle from patients who were previously sedated. The procedure was performed via colonoscopy. The tissue was postfixed for 24 h in fixative solution at 4 °C. For microbiome analysis, tissue samples were snap frozen at -70ºC. For immunohistochemistry tissue samples were cryoprotected using increasing concentrations of sucrose in PBS (10, 20 and 30%) and embedded in optimal cutting temperature (O.C.T.) (Tissue Tekª, ThermoFisher) as described previously [4]. Sections were cut at 20 µm of thickness on a cryostat (Cryostar NX50, ThermoScientific) at − 20 °C and mounted on SuperFrost© microscope slides (Thermofisher).

All procedures were approved by the Ethics Committees of the Faculty of Medicine and the University Hospital of University of Coimbra, and all patients and controls signed the informed consent report.

### Animal model and experimental design

A total of 115, 32-week-old (adult) C57BL/6 male mice, divided during time into eight different cohorts, were used in this study in (46 untreated; 23 were colonized with healthy donor fecal material (HC mice) and 46 were colonized with PD patient fecal material (PD mice). Mice were purchased from Charles River (Barcelona, Spain) and housed in our animal colony (Animal Research Center, University of Coimbra), under controlled light (12 h day/night cycle), temperature and humidity (45–65%), with free access to standard hard pellet chow and water. Signs of distress were carefully monitored and although none occurred, a rapid decrease in body weight > 15–20% was defined as a potential humane endpoint for the study. The EU and Portuguese legislation (Directive 2010/63/EU; DL113/2013, 7 August) for the care and the use of animals was followed. All procedures were in accordance with the ethical standards of the Animal Welfare Committee of the Center for Neuroscience and Cell Biology and the Faculty of Medicine, University of Coimbra and the researchers received adequate training (FELASA certified course) and certification from the Portuguese authorities (Direção Geral de Veterinária) before performing the experiments.

**Fig. 1:**
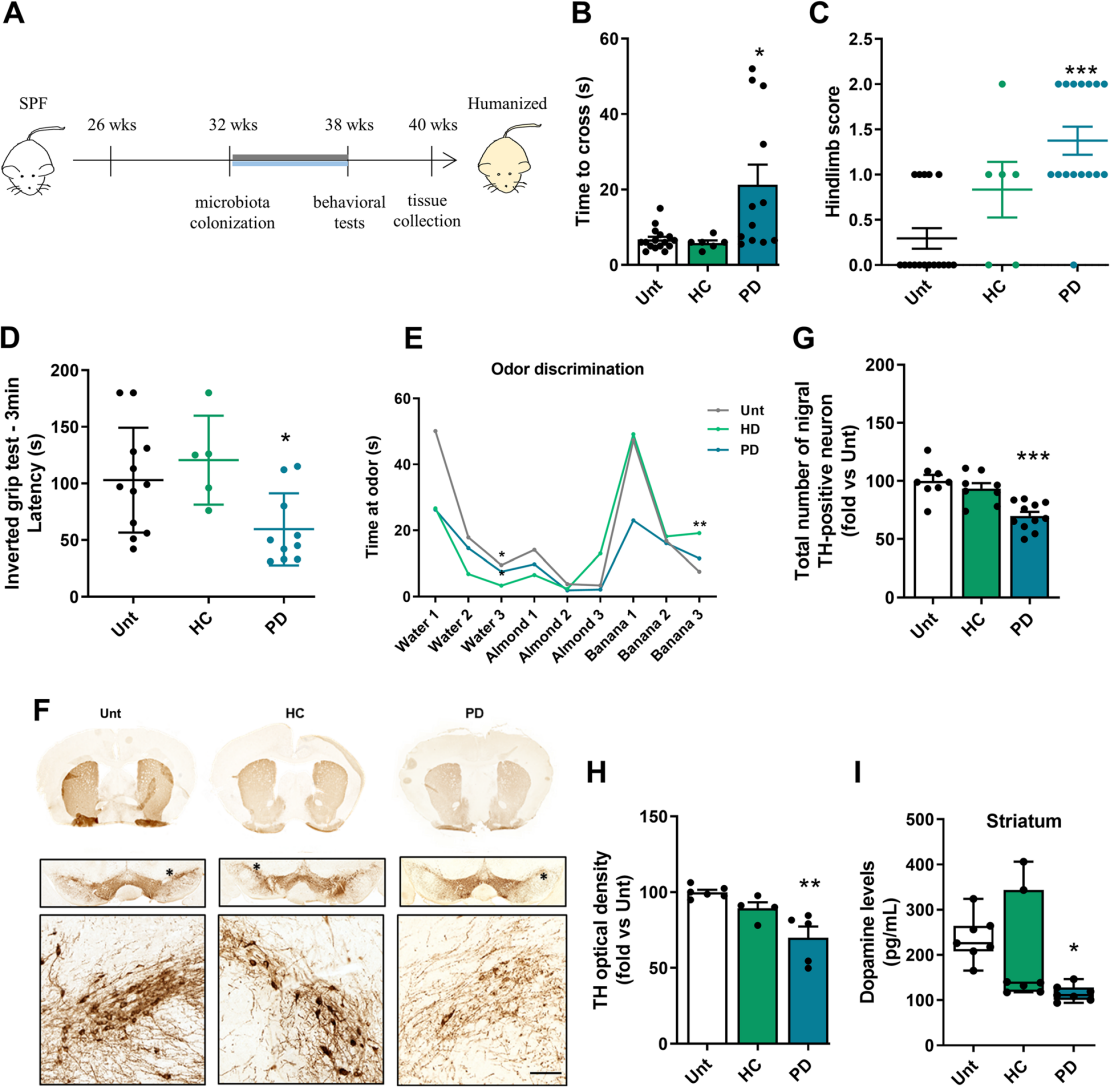
Dopaminergic neurodegeneration and motor alterations in WT mice transplanted with fecal microbiota. **A** Schematic representation of experimental design (**B**) Balance and motor coordination performance were assessed with the beam walking test (*n* = 6–16 mice per group). **C** Hindlimb clasping reflex was monitored, as a quick phenotypic neurological scoring system to assess disease progression (*n* = 6–17 mice per group). **D** The inverted grip test was used to assess of limb muscle strength (*n* = 4–12 mice per group). **E** Odor discrimination and habituation was tested in time spent (s) non**-**social odors (*n* = 6–8 mice per group). **F** Representative photomicrographs of brain coronal sections immunostained for TH.^+^ in the striatum (STR) and substantia nigra (SN). **G** Total number of nigral TH-positive neurons in the SN assessed by stereological analysis (*n* = 8–11 mice per group). **H** OD analysis of the TH-positive fibres in the STR group normalized to the untreated group (*n* = 4–6 mice per group). **I** Striatal dopamine levels in pg/mL (*n* = 7 mice per group). *p < 0.05, **p < 0.01, ***p < 0.001, using one-way ANOVA with Dunnet´s test (**G** and **H**) or Kruskal–Wallis with Dunn´s test (**B**-**D** and **I**). Data are expressed as mean ± SEM. Scale bars are 100 µm (magnified inner square) and 1 mm. See also Tables S1-S2 and Figures S1-S4

To determine the impact of gut dysbiosis on the development and progression of PD pathology, we transplanted fecal material from 5 PD patients and 4 age-matched healthy donors into wild-type male C57BL/6 mice (each mouse received microbiota from a single donor). Seminal experiments were conducted utilizing samples from these 9 donors to substantiate the efficacy of fecal microbiota transplantation (FMT) from PD patients' gut microbiomes as a significant instigator of pathology. Individual WT mice colonized with microbiomes from either 4 healthy control (HC) individuals or 5 Parkinson's disease (PD) patients were used to assess various parameters: motor behavior, midbrain TH + neurons, ileum**-**associated mucosa SFB percentage, ileum CD11b + cells, ileum ZO**-**1 levels, ileum aSyn aggregates (Figure S3), ileal levels of TNF, IL**-**6, and IL**-**17, as well as midbrain levels of IL**-**1β and IL**-**17. The remaining experiments utilized mouse tissues sourced from healthy control donor HC1 and Parkinson's disease donor PD1. Mice were generated by colonizing 32**-**week**-**old wild-type animals with PD or HC fecal material, administered in gelatin once a day during the first 1 week and twice a week for 5 weeks (Fig. 1A). Additionally, we performed a longitudinal study to determine temporal propagation of pathology using 4 Unt and 5 PD mice after 1**-**week colonization (33 wks.); 4 Unt and 5 PD mice after 2-weeks colonization (34 wks.); 5 Unt and 5 PD mice after 3**-**weeks colonization (35 wks.) and 3 Unt and 3 PD mice after 4-weeks colonization (36 wks.) (Fig. 9A).

Body weight was monitored twice a week throughout the study. Animals were also weighed immediately before euthanasia. Results were expressed in terms of body weight (g). Immediately after euthanasia, whole blood was collected from selected animals to determine occasional blood glucose levels by the glucose oxidase reaction, using a glucometer (Glucometer-Elite, Bayer SA, Portugal) and compatible strips. Results were expressed as mg glucose/dL blood. At the end of the experiments (38 weeks), fecal pellets were collected from animals individually housed in a clean cage.

### Microbiome profiling

Human fecal material and terminal ileum mucosa biopsies, mouse fecal pellets and terminal ileum mucosa-associated material were collected for microbiome profiling. The mouse's ileum was extracted, flushed with sterile PBS, sectioned into pieces, and the luminal area was gently scraped and frozen. Human ileal biopsies were frozen. Microbial genomic DNA from frozen fecal and ileal samples was extracted using the NZY Soil gDNA Isolation Kit (NZYTech, Portugal), which includes a mechanical lysis step (with beads). The amount and quality of the extracted genomic DNA was assessed in a Nanodrop 2000 (Thermo Scientific). DNA integrity was assessed by PCR using universal primers for the 16S rRNA gene [27 F (5’-GAGTTTGATCCTGGCTCAG-3’) and 1525 R (5’-AGAAAGGAGGTGATCCAGCC-3’)] as previously described [39]. Total microbial DNA was sequenced at Genoinseq sequencing facilities (Cantanhede, Portugal) or at Novogene (UK) using the Illumina MiSeq® platform (Illumina, USA). Universal forward primer 515F-Y (5’- GTGYCAGCMGCCGCGGTAA-3’) and reverse primer 926R (5’-CCGYCAATTYMTTTRAGTTT-3’) [40] were used to target the V4-V5 hypervariable region using a standard protocol. Raw data processing, clustering and taxonomic annotation were performed using the mothur package version 1.44.1 (www.mothur.org) [41] and Silva reference files, release 138 [42]. To perform a comprehensive meta-analysis of the microbiome data, including community profiling and differential abundance, R (v 4.0.4) and the tidyverse package (v 1.3.0) were used to manipulate and visualize the data [42], while compositional analysis of the microbiota was performed using the phyloseq [42] and microbiome packages. Alpha diversity was measured on unfiltered data using the Shannon index, with statistical significance being assessed using the Mann–Whitney test and the Nemenyi test for pairwise comparisons, with Benjamini–Hochberg correction. For the remaining analysis, genera not detected in more than 1/3 of each sample group of samples were filtered. For beta diversity assessment, a non-metric multidimensional scaling (NMDS) analysis based on the Bray–Curtis dissimilarity index was used to visualize community-level similarity. Uneven sequencing depth in ileum samples was corrected by standardization to the median sequencing depth, and permuted analysis of variance (PERMANOVA) was used to assess statistical significance. Pie charts were generated from data transformed to relative abundances, after filtering for low prevalence (retaining only the genus present in 1/3 of the samples). Filtered data were also used to perform differential abundance analysis, with fold-change calculation and statistical assessment performed using the DESeq2 Wald test algorithm [42].

### Behavior

At the end of the treatments, motor activity, anxiety-like behavior, spatial memory and olfactory function were assessed in our animals, as previously described [4]. All tests were performed under red light, in a sound-attenuated observation room where the mice had been habituated for at least 1 h. The apparatus was cleaned with 10% ethanol between animals.

Balance and subtle alterations in motor coordination, limb strength of the mice and severity of motor dysfunction were assessed using the beam walking, the inverted grid and the hindlimb clasping tests, respectively. Briefly, in the beam walking test, mice were allowed to traverse a narrow beam (8 diameter beam) to reach an enclosed safety platform in two consecutive trials, with a maximum time per trial of 90 s. Any animal that did not cross within the allotted time was assigned a maximum score of 90 s for analysis. For the grip test, each mouse was placed in the center of a metal grid and turned upside down so that the mouse was hanging and clinging to the grid (40 cm above the floor and with soft padding to cushion falling). The mice were allowed to move freely on the underside of the grid during a maximum “cling time” of 90 s. As an indicator of motor deficits, disease progression and neurodegeneration, the posture of the mice was assessed in the hindlimb clasping test. The tail of the mouse was grasped near its base and the animal was lifted away from all surrounding objects. The hindlimb position was then observed for 10 s and scored. A score between 0 and 3 was assigned depending on the clasping reflex observed for each mouse: a score of 0 was assigned if the hindlimbs were consistently extended away from the abdomen; a score of 1 was assigned if one hindlimb was partially retracted toward the abdomen for more than 50% of the time; a score of 2 was assigned if both hindlimbs were partially retracted toward the abdomen for more than 50% of the time; a score of 3 was assigned if both hindlimbs were completely retracted for more than 50% of the time.

Evaluation of locomotion, anxiety and stereotypical behaviors in mice was carried out with the open field test, through the analysis of the parameters of percentage of time resting and time spent in the center of the arena, number of feces expelled in the experimental time, mean velocity of mice and total distance travelled. This test used an Acti**-**Track System (PanLab, Barcelona, Spain) to track the activity of mice, for 30 min, in a 50 cm wide × 50 cm deep × 50 cm high arena.

Short-term spatial memory was assessed using the T-maze test (30 cm long × 10 cm wide × 20 cm high). The T-maze spontaneous alternation test was chosen because of its simplicity and minimal stress on the animals. At the beginning of each run, mice were placed at the start arm (bottom of the "T") and given 2 min to choose the right or left target arm. Once committed to a particular target arm (all four paws entered the arm), the "T" junction between the start arm and the opposite target arm was blocked to prevent the mice from entering the opposite target arm. The mice were allowed to explore the target arm for 30 s. With the "T" junction block removed, the mice were returned to the start arm and allowed to choose a target arm. An alternation was defined as the mouse entering an arm opposite to the one it entered on the previous run. Mice completed 5–6 trials. The time taken to choose one arm was also scored.

To investigate olfactory ability and the ability to discriminate between stimuli (odors), we used the Odor Discrimination and Habituation Test. In this non**-**invasive, spontaneous behavioral task, mice were presented to one of five odors (water and almond and banana extracts (non-social odors) and two social odors (obtained by swabbing the outside of two different cages of same**-**sex mice from this study) using a saturated cotton-tipped wood applicator (located on the bottom of the cage, 1 cm above the cage floor). Each odor was presented three times in a row. Mice with their nose within 2 cm of the applicator tip were considered to be exploring the odors. The evaluation consisted of a cross-habituation phase: time spent sniffing the applicator with a novel odorant stimulus (mice spontaneously recognize new odors) versus a habituation phase: time spent on the repeated stimulus.

### Immunohistochemistry, immunofluorescence and microscopy analyses

Histological samples and post-mortem microscopic analyses were performed as described in previous studies [4, 43]. Briefly, tissue samples were collected after transcardial perfusion with saline (0.9% NaCl) followed by 50 mL of fixative solution (4% paraformaldehyde (PFA) and 0.1% glutaraldehyde in PBS). Brains and intestines were postfixed in fixative solution for 24 h at 4 °C. The intestines were previously rinsed with PBS and cut into 1 cm pieces. Tissue samples were cryoprotected using increasing concentrations of sucrose in PBS (10, 20 and 30%) as previously described [4, 43]. Coronal sections were cut at 20 µm of thickness on a cryostat (Cryostar NX50, ThermoScientific) at − 20 °C and mounted on SuperFrost© slides (Thermofisher).

In the gut samples, we determined CD11^+^ cell enrichment, ZO-1 integrity scoring, mitochondrial morphology of enteric neurons, aSyn oligomers and the percentage of Th17 cells (CD4^+^/IL**-**17^+^) were determined by immunofluorescence. CD4^+^ infiltration, aSyn and phosphorylated-aSyn (p-aSyn) expression by immunohistochemistry. The immunofluorescence method was performed as previously described [4]. Briefly, samples were incubated with rabbit anti-ZO-1 (Abcam, 1:300), mouse anti-CD11b (BioRad, 1:200), rabbit anti-CD4 (Cell Signaling, 1:200), rabbit anti-TOM20 (Santa Cruz, 1:400), mouse anti-β3-tubulin (Cell Signaling, 1:200), rabbit anti-aSyn aggregate (Abcam, 1:300) or mouse FITC-conjugated anti-IL-17 (Santa Cruz Biotechnology, 1:50) in PBS containing 1% donkey or goat serum and 0.25% Triton-X-100 for 24 h at 4° C. For mouse primary antibodies on mouse tissue, M.O.M. mouse Ig blocking reagent was applied 1 h prior to the blocking step. Secondary antibodies were donkey or goat anti-rabbit, or anti-mouse conjugated to Alexa Fluor 488 or Alexa Fluor 594 (Life Technologies, 1:250). Sections were stained with Hoechst 33342 (Sigma, 1:1000) before mounting with Mowiol© (Sigma). Immunofluorescence images were captured using an LSM710 (Zeiss) confocal microscope with different magnification objectives (10 × , 20 × and 40 ×) at a resolution of 1024 × 1024.

#### Immunofluorescence

For intestinal barrier integrity, the analysis was scored using a grading scale as previously described [4, 44, 45]. Between 7–10 images with 3–5 villi per image and animal were randomly acquired and blindly scored. b) For CD11b^+^ scoring, ten images were randomly acquired per animal, a total of 304 villi were analyzed (21–24 villi per animal), the number of CD11b^+^ cells was counted and divided by the total counting area (mm^2^). c) Mitochondrial network analysis was performed by TOM20 expression detected by immunofluorescence at 63 × magnification using an LSM710 microscope. Network parameters were obtained using an ImageJ macro MiNA [46] applied on at least five βIIITubulin-positive cells (outlined using the square tool) per image in tissue sections, and applied on total image in tissue sections. d) aSyn quantification was conducted using ImageJ, wherein the percentage of submucosa/muscularis propria area occupied by aSyn aggregates was measured. e) To calculate the percentage of Th17 cells (CD4^+^/IL-17^+^), ten images were taken randomly per animal. The number of CD4^+^ cells expressing IL-17 was expressed as a percentage, considering the total of CD4^+^ cells as 100%. Human biopsies were analyzed using the same criteria, but images were taken at 40 × magnification. Between 7–10 images with 2–4 villi per image and sample were randomly acquired and blindly scored.

#### Immunohistochemistry

Immunohistochemistry was performed as previously described [4, 43]. Briefly, sections were thawed, hydrated, treated for antigen retrieval, quenched and blocked. For mouse primary antibodies on mouse tissue, M.O.M. mouse Ig blocking reagent was applied 1 h prior to the blocking step. We determined: a) the number of CD4^+^ cells divided per the total counting area (mm^2^), b) the optical density (OD) of aSyn expression and c) the number of p-aSyn positive cells with rabbit anti-CD4 (Cell Signaling, 1:200), rabbit anti-aSyn (Abcam, 1:500) or mouse anti-p-aSyn (WAKO, 1:500) in PBS containing 1% goat or horse serum and 0.25% Triton-X-100 for 24 h at 4 °C. Secondary antibodies biotinylated goat anti-rabbit or anti-mouse IgG (Vector, 1:200) were diluted in PBS containing 0.25% Triton-X-100, followed by incubation with the avidin/biotin complex-HRP (VECTASTAIN Elite ABC Kit Standard, Vector Laboratories, CA, USA) for 30 min. Finally, the sections were counterstained with 1% cresyl violet. The tissue was dehydrated and mounted on DPX mounting medium (Sigma). CD4^+^ cell counts were estimated using the optical fractionator method in combination with the dissector principle and unbiased counting rules [47]. Between 4–5 coronal sections of the ileum were analyzed using Stereo Investigator software (MBF Bioscience) attached to an Axio Imager Z2 microscope (Zeiss). CD4^+^ cells were counted at 40 × magnification (1.4 numerical aperture, oil immersion). The grid size was 250 × 250 µm and the counting frames were 150 × 150 µm. The coefficient of error was calculated according to Gundersen and coworkers [47]. An error of CE < 0.1 (m = 1 class) was accepted for the analysis.

The level of aSyn expression was assessed by OD. Between 7–9 sections per animal were captured at 20 × magnification using the AxioScan slide scanner (Zeiss) and color deconvoluted using the “Color Deconvulation” plugin (https://imagej.net/Colour_Deconvolution) to measure the OD of DAB staining. OD was measured using ImageJ software (version 1.40 National Institute of Health). Images were converted to 8-bit grayscale and the mean intensity was quantified. Values were converted from pixels to OD using the Kodak No. 3 Calibrated Step Tablet template as a pattern curve. To assess the number of p-aSyn^+^ cells, 4 random images of coronal sections of the ileum were taken and quantified the number of p-aSyn^+^ cells in the entire section was quantified by counting the presence of intracellular staining. Four sections per animal were used. In human samples, the number of p-aSyn^+^ cells were quantified using a 150 × 150 µm counting frame in 10 different areas per Sect. (5 sections per sample).

In brain samples, immunofluorescence and immunohistochemistry protocols were performed as described above, except for a) IgG staining, which is a direct immunohistochemistry using biotinylated anti-mouse IgG (Vector, 1:1000). By immunofluorescence, we determined: b) the expression of Trem2 in Iba1^+^ cells in the SN and c) the estimated number of TH + and choline acetyltransferase positive (ChAT +) cells in the dorsal motor nucleus of the vagus nerve (DMV); and by immunohistochemistry, we assessed d) the expression of CD4 + in the SN; e) aSyn in STR, SN, DMV and CX and f) the expression of TH in STR by measuring the immunoreactivity by OD. We also performed g) the stereological quantification of TH + cells in the SN to determine the degree of dopaminergic neurodegeneration and the number of perivascular IgG-immunopositive staining per total area (mm^2^) in the cortex, striatum (STR) and SN to assess BBB integrity. Primary antibodies were rabbit anti-CD4 (Cell Signaling, 1:200), rabbit anti-Iba1, (Wako, 1:500), sheep anti-Trem2 1:200, R&D Systems), rabbit anti-TH (Millipore, 1:300), mouse anti-ChAT (ThermoFisher Scientific, 1:200), rabbit anti-aSyn (Abcam, 1:500 or mouse anti-p-aSyn (WAKO, 1:500). Secondary antibodies were donkey or goat anti-rabbit Alexa Fluor 488 (Abcam, 1:500), donkey anti-sheep Alexa Fluor 647 (Abcam, 1:500), goat anti-mouse Alexa Fluor 488 or anti-mouse Alexa Fluor 594 (Molecular Probes, 1:500), for immunofluorescence or biotinylated goat anti-rabbit or anti-mouse IgG (Vector, 1:200) for immunohistochemistry.

To assess the expression of Trem2 in Iba1^+^ cells in the SN, images of identical regions were acquired using a confocal microscope LSM710 (Zeiss) with a Plan-Apochromat 40 × /1.4 Oil DIC M27 objective at a resolution of 1024 × 1024. A total of six images per animal were randomly acquired across three different sections of the SN. Z-stacks were converted to maximum projection images using Fiji image software. Images were thresholded using the Triangle algorithm. To quantify the % area of Trem2 contained in Iba1^+^ cells, images were split into red and green channels and were converted to 8-bit images. To create a binary mask, a threshold was applied to both images to remove the background. The mask of the green channel (Iba1) was overlapped with the red mask (Trem2) and the ratio (%) of the red area (Trem2) within the green area (Iba1) was calculated. This was done using Fiji image software and the acquisition and analysis were performed blindly.

To determine TH immunoreactivity in the striatum, slides were scanned at 20 × magnification using the AxioScan slide scanner (Zeiss). A total of eight coronal sections systematically distributed along the anteroposterior axis of the striatum, with an evaluation interval of ten per animal, were quantified. OD was measured as described above. In this case, however, background staining was corrected by subtracting values obtained from adjacent cortical areas.

The number of TH + cells in the SN was estimated by stereological analysis as described above. In this case, a total of eight sections systematically distributed along the anteroposterior axis of the SN, with an evaluation interval of seven per animal, were included in the counting procedure. TH-positive cells were counted using a 40 × magnification objective (1.4 numerical aperture, oil immersion). The grid size and the counting frames were the same as described above. The coefficient of error was calculated according to Gundersen and coworkers [47]. An error of CE < 0.1 (m = 1 class) was accepted for the analysis.

To determine the expression of aSyn and the number of p-aSyn positive cells in the SN and DMV, we performed the same analysis as described above for the ileum samples. For the DMV and SN, between five and eight coronal sections systematically distributed along the anteroposterior axis, were quantified with an evaluation interval of five and seven per animal, respectively.

IgG immunostaining was performed as direct immunohistochemistry as it has been described in previous studies [4, 43]. Briefly, slides were scanned at 20 × magnification using the AxioScan slide scanner (Zeiss). The number of brain microvascular vessels with blood–brain barrier disruption was quantified in the cortex, STR and SN. Eight coronal sections systematically distributed along the anteroposterior axis were stained and quantified with an evaluation interval of ten per animal for cortex and striatum, and eight for SN. To assess BBB integrity, we quantified the number of microvascular leakages (IgG immunopositive staining in the perivascular area) per total area (mm^2^).

### Plasma and peripheral blood mononuclear cells (PBMCs)

Twenty milliliters of venous blood was collected by venipuncture in K2- EDTA-containing tubes from both PD and disease-free HC individuals. Erythrocytes, plasma and PBMCs were isolated by Ficoll-Histopaque density gradient centrifugation, according to the manufacturer’s instructions. Briefly, blood samples were diluted with equal volume of Hanks’s balanced salt solution (HBSS), layered on Ficoll-Histopaque and centrifuged at 300 × g for 30 min at room temperature. Mouse blood was collected by cardiac puncture using a 23G needle syringe in animals previously deeply anesthetized with sodium pentobarbital (150 mg/kg) and placed in tubes coated with EDTA (0.5 M). Blood samples were transferred to 15 mL tubes containing Histopaque© 1083 solution (Sigma) and diluted (1:1) in phosphate-buffered saline (PBS). Tubes were centrifuged at 400 × *g* for 30 min at RT. PBMC halo and plasma were carefully collected with a Pasteur pipette and transferred to new tubes. PBMC halo in tubes containing 5 mL PBS were washed twice with PBS and centrifuged at 250 × *g* for 10 min at 4 ºC. Pellets of centrifuged PBMC halos were resuspended in lysis buffer, followed by three cycles of freezing and thawing in liquid nitrogen. Lysed samples were then centrifuged at 17,968 × *g* for 10 min, at 4ºC. The protein content of the resulting supernatants was determined using the Pierce™ BCA Protein Assay Kit.

### Flow cytometry

PBMC pellet was incubated with anti-mouse CD45 PerCP (clone 30F11), anti-mouse CD3 FITC (clone REA641), anti-mouse CD4 APC (clone REA604) and anti-mouse CD8 PE (clone REA601) (1/50) (Miltenyi biotec) for 10 min at 4 ºC. The cell suspension was washed with PBS, centrifuged at 250 × *g* for 10 min at 4 ºC and the pellet was fixed with 2% PFA solution for 10 min at 4 ºC. Finally, the cells were centrifuged at 250 × *g* for 10 min at 4 ºC and the pellet was resuspended in PBS and analyzed by flow cytometry. The analysis was performed on the BD FACSCalibur cytometer (BD Bioscience), which was pre-set with voltage adjustments, compensated using single-stained cells, and the true background level was defined using the isotype control antibodies Rat Anti-IgG2a PerCP, REA Control-FITC, REA Control-PE and REA Control-APC (Miltenyi Biotec). The gating strategy was performed using FlowJo© software (BD Bioscience). More than 10,000 events were acquired in the region of interest (ROI), identified as the lymphocyte area in the forward versus side scatter dot plot. The percentage of CD4 and CD8 was obtained by gating the CD45^+^CD3^+^ events contained in the ROI.

### Western blotting, spectrophotometry and ELISA determinations in brain and intestinal homogenates

At the end of the behavioral tests, mice were deeply anesthetized with isoflurane before being euthanized by cervical dislocation for tissue isolation. Specifically, the midbrain, striatum and ileum were isolated and immediately snap frozen and stored at − 80 °C until further analysis. For Western blotting, spectrophotometry and ELISA analysis, ileum, duodenum, jejunum and cecum were homogenized as follows: 1 cm pieces were cut, washed in ice-cold PBS and homogenized in hypotonic lysis buffer (0.1% Triton X-100, 25 mM HEPES, 2 mM MgCl_2_, 1 mM EDTA and 1 mM EGTA, pH 7.5) supplemented with 2 mM DTT, 0.1 mM PMSF and a 1:1000 dilution of a protease inhibitor cocktail. Samples were then frozen three times in liquid nitrogen, sonicated on ice (3 pulses) and centrifuged at 17,968 × *g* for 10 min at 4 ºC. Supernatants were collected and protein content was determined using the Pierce™ BCA Protein Assay Kit (Thermo Scientific, Rockford, IL, USA) according to the manufacturer’s instructions for the plate reader. For Western blotting, mesencephalic tissue was homogenized in hypotonic lysis buffer (10% Triton X-100, 25 mM HEPES, 2 mM MgCl_2_, 1 mM EDTA and 1 mM EGTA, pH 7.5) supplemented with 2 mM DTT, 0.1 mM PMSF, 2 mM sodium orthovanadate, 50 mM sodium fluoride and a 1:1000 dilution of a protease inhibitor cocktail from Sigma (St. Louis, MO, USA). Samples were then frozen three times in liquid nitrogen, centrifuged at 20,000 × *g* for 10 min at 4ºC and the supernatants collected. For caspase-1 determination and ELISA kits, mesencephalic tissue was homogenized in 0.1% Triton X-100 containing hypotonic lysis buffer (10 mM HEPES; 3 mM MgCl_2_; 1 mM EGTA; 10 mM NaCl, pH 7.5), supplemented with 2 mM DTT, 0.1 mM PMSF and a 1:1000 dilution of a protease inhibitor cocktail from Sigma (St. Louis, MO, USA). Samples were then incubated on ice for 40 min and centrifuged at 2,300 × *g* for 10 min at 4 ºC. Supernatants containing the cytosolic fraction were collected. For the determination of dopamine levels, striatal tissues were sonicated in ice-cold 0.2 M perchloric acid, centrifuged at 13000 rpm, 7 min, 4 °C. Supernatants were collected while pellets were resuspended in 1 M NaOH. Protein content was determined using the Pierce™ BCA Protein Assay Kit (Thermo Scientific, Rockford, IL, USA) according to the manufacturer’s instructions for the plate reader.

For the analysis of TLR4 and pro-IL-1β tissue lysates were loaded on SDS-PAGE gels under reducing conditions (resuspended and boiled for 5 min at 95ºC in 4 × Tris–Cl/SDS, pH 6.8, 30% glycerol, 10% SDS, 0.6 M DTT, 0.012% bromophenol blue). For the analysis of aSyn oligomers tissue lysates were loaded onto PAGE gels under non-reducing and non-denaturing conditions (suspended in 40% glycerol, 2% SDS, 0.2 M Tris–HCl pH 6.8, 0.005% Coomassie Blue). After electrophoresis, the samples were transferred to PVDF membranes (Millipore, Billerica, MA, USA) and after transfer the membranes were blocked for 1 h in Tris-buffered solution (TBS) containing 0.1% Tween-20 and 3% BSA. The membranes were then incubated with the appropriate primary antibodies at 4 °C with gentle agitation: 1:100 anti-TLR4 from Santa Cruz Biotechnology (Santa Cruz, CA, USA); 1:500 anti-pro-IL1β from Santa Cruz Biotechnology (Santa Cruz, CA, USA); 1:1000 polyclonal anti-aSyn, from Cell Signaling (Danvers, MA, USA). Membranes were reprobed with 1:1000 β-III tubulin from Cell Signaling (Danvers, MA, USA) to confirm equal protein loading. After incubation with the primary antibody, the membranes were washed three times with TBS containing 3% BSA and 0.1% Tween (5 min each time) and then incubated with the appropriate horseradish peroxidase-conjugated secondary antibody for 2 h at RT with gentle agitation. Membranes were washed three times and bound antibodies were detected by developing with an alkaline phosphatase enhanced chemical fluorescence reagent (ECF from GE Healthcare, Piscataway, NJ, USA). Fluorescence signals were detected using a Biorad Chemidoc Imager. Analysis of Western blot band densities was performed using Quantity One software (Bio-Rad).

To assess caspase-1 activation, 40 μg of tissue lysates were incubated with 100 μM of the colorimetric substrate for caspase-1 (Sigma Chemical Co., St. Louis, MO, USA) in reaction buffer (25 mM HEPES pH 7.5, 0.1% (w/v) 3-[(3-cholamidopropyl) dimethylammonio]-propanesulfonic acid (CHAPS), 10% (w/v) sucrose, 2 mM DTT) for 2 h at 37 °C, protected from light. Enzymatic cleavage of the substrate was detected at 405 nm using a Spectramax Plus 384 spectrophotometer (Molecular Devices, Sunnyvale, CA, USA).

Ileum, midbrain lysates and blood (25 μg) were used to determine inflammatory markers using ELISA kits, specifically TNF, IL-17, IL-10, IL-8, IL-6, IFNγ, IL-1β, and NFκB p65, according to the manufacturer’s instructions. Jejunum, duodenum and cecum lysates were used to determine IL-17 levels using an ELISA kit. Absorbance was detected at 450 nm using a SpectraMax Plus 384 multiplate reader. Results are expressed as μg/mL protein for NFκB p65 and as pg/mL for the other markers.

STR homogenates (50 μL) were used to calculate dopamine levels using an ELISA kit, according to the manufacturer’s instructions. Absorbance was detected at 450 nm in a SpectraMax Plus 384 multiplate reader. Results are expressed as pg/mL.

### Mitochondria isolation and oxygen consumption rate (OCR)

Fresh mitochondria were isolated from mesencephalic and cortical areas using a discontinuous Percoll density gradient centrifugation as previously described by Ferreira and co-workers [48]. Small pieces from each area were isolated and washed in ice-cold isolation buffer (225 mM mannitol, 75 mM sucrose, 1 mM EGTA, 5 mM HEPES, pH 7.2/KOH). Mesencephalic and cortical mitochondria were then homogenized with 25 up and down strokes in a Dounce All-Glass Tissue Grinder (Kontes Glass Co., Vineland, NJ, USA) using pestle A (clearance: 0.07–0.12 mm) followed by 25 up and down strokes with pestle B (clearance: 0.02–0.056 mm). The tissues were then briefly centrifuged at 1100 × *g* for 2 min at 4 °C. Supernatants were collected and mixed with fresh ice-cold 80% Percoll solution prepared in 1 M sucrose, 50 mM HEPES, 10 mM EGTA, pH 7.0, then carefully layered on the top of fresh ice-cold 10% Percoll solution and further centrifuged at 18,500 × *g* for 10 min at 4 °C. The mitochondrial-containing pellet was then gently resuspended in 1 mL washing buffer (250 mM sucrose, 5 mM HEPES–KOH, 0.1 mM EGTA, pH 7.2). The mitochondrial-containing fractions were centrifuged again at 10,000 × *g* for 5 min at 4 °C and the final mitochondrial pellet was resuspended in ice-cold washing buffer. Protein content was determined using a Bio-Rad protein assay. 5 µg of fresh mesencephalic or cortical mitochondria were used for OCR measurements using a Seahorse XF24 Extracellular Flux Analyzer (Seahorse Bioscience, Billerica, MA, USA). Mitochondria were centrifuged at 2,200 × *g* for 20 min at 4 °C in a 24-well XF culture plate precoated with 1:15,000 polyethyleneimine (PEI) solution in mitochondrial assay solution (MAS: 70 mM sucrose, 220 mM mannitol, 10 mM KH_2_PO_4_, 5 mM MgCl_2_, 2 mM HEPES, 1 mM EGTA, pH 7.2) to adhere to the bottom of the plate [48, 49]. Isolated mitochondria were then incubated in MAS containing succinate (10 mM; Complex II substrate) plus rotenone (2 mM; Complex I inhibitor) for 8 min at 37ºC in a CO_2_-free incubator and then the plate was transferred to the Seahorse XF24 flux analyzer. First, 4 mM ADP was added to energize the mitochondria and then 2.5 μg/mL of oligomycin (inhibitor of ATP synthase) was added to prevent respiration derived from ATP synthesis. Next 4 μM of the uncoupler FCCP was added, which caused an increase in OCR reflecting the maximum respiratory chain activity as well as the maximum substrate oxidation rate. Finally, 4 μM of antimycin A (Complex III inhibitor) was added to block the respiratory chain and the remaining OCR.

The following determinations were calculated as previously described [48]: basal respiration: last rate measurement before first injection; maximal respiration: last rate measurement after FCCP injection; ATP synthesis: last rate measurement before oligomycin injection minus minimum rate measurement after oligomycin injection.

### Statistical analysis

Microbiome population statistics are described in detail in the Microbiome Profiling section above. Statistical analysis of the datasets was performed using GraphPad Prism 8 software (GraphPad Software, San Diego, CA, USA). All data are presented as mean ± SEM. Normality analysis (Shapiro–Wilk’s test) was used to determine the subsequent parametric or non-parametric tests. Pairwise comparisons were performed using unpaired Student’s t-test or Mann–Whitney test. Multi-group comparisons were performed using one-way ANOVA followed by Dunnet’s post-hoc test or Kruskal–Wallis test followed by Dunn’s post-hoc test. All statistical tests were two-tailed, and the significance values were annotated as follows: *p < 0.05, **p < 0.01, ***p < 0.001. P and N values are given in each figure legend.

## Results

### Fecal microbiota from PD patients colonizes WT mice and induce loss of mesencephalic TH^+^ cells and motor behavior deficits

Several studies have demonstrated an association between gut dysbiosis and PD, and some have used FMT to induce beneficial and detrimental effects in the host [3, 50–55]. In our study, the fecal microbiomes of 44 PD patients and 21 HC (Table S1), were profiled using high-throughput next-generation sequencing on the Illumina MiSeq® platform.

In human fecal material, we detected 52,849.33 ± 10,650.39 reads per sample, and sequences for 172 bacterial genera, 68 families, 40 orders, 17 classes, and 9 phyla. We observed a non-statistically significant decrease in alpha diversity of bacterial communities (Figure S1A) and a similar overall community composition (beta diversity; Figure S1B) between PD patients and HC. We found that the relative levels of 2 genera were differentially abundant and increased significantly in the fecal microbiota of PD patients, namely *Lactobacillus* and *Streptococcus* (Figure S1C**, **D). We observed a non-statistically significant increase in *Bifidobacterium* and *Akkermansia* (Figure S1E**, **F). Our results also showed a significant decrease in the relative abundance of 1 genus: *Faecalibacterium* and a non-statistically significant decrease in *Roseburia,* and *Prevotella* (Figure S1G**-**I).

To confirm that the PD phenotype can be induced by the physical transfer of the gut microbiome from PD patients to a host, we collected, preserved, and transplanted 5 PD and 4 HC fecal microbiota in commercial gelatin into different WT C57BL/6 male mice (Table S2). Treatments had no effects on body weight and glycaemia in all the experimental groups (Figure S2A**, **B).

**Fig. 2:**
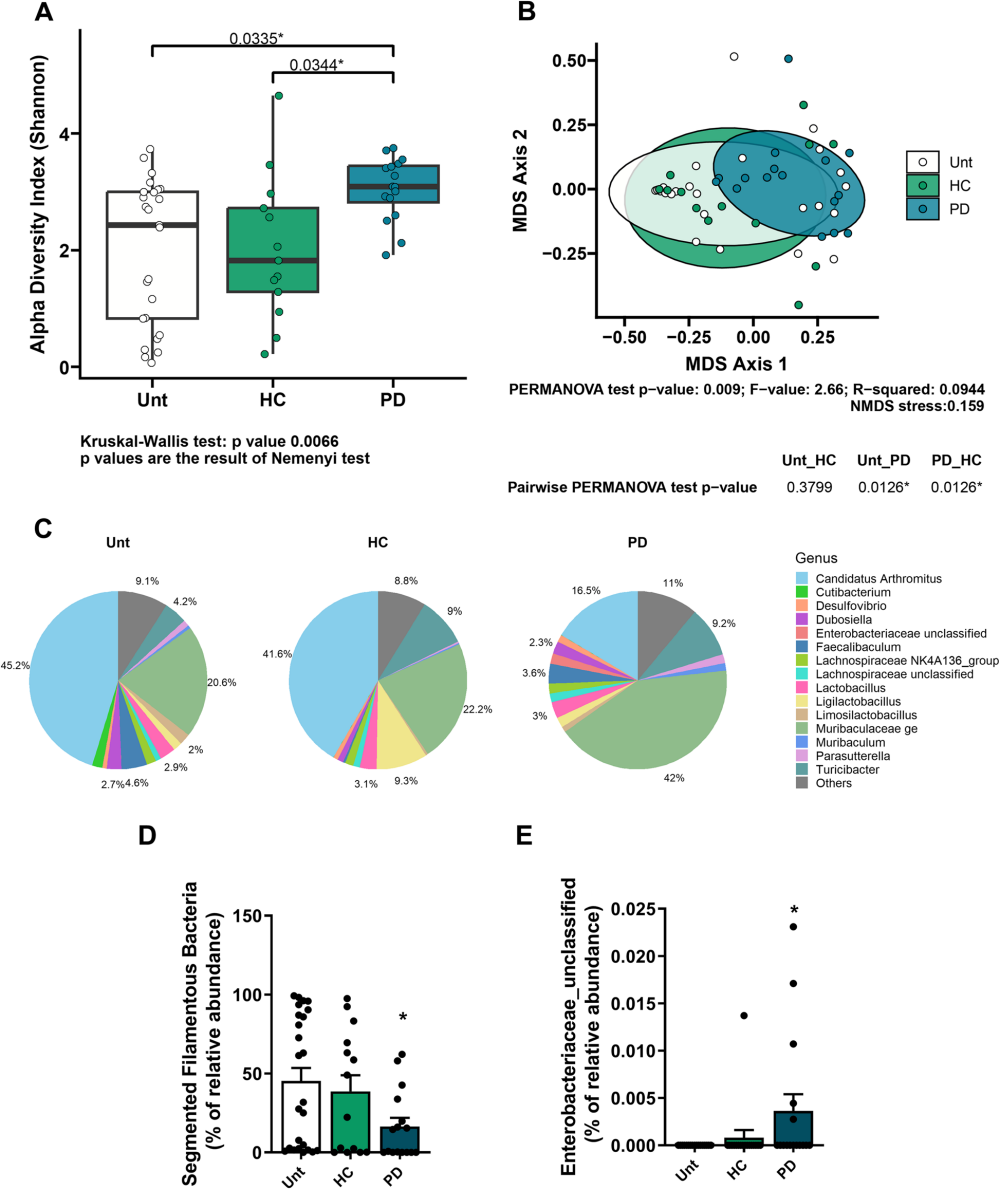
Microbiota associated with the ileal mucosa of mice. **A** Alpha diversity measured using the Shannon index at the OTU level derived from 16S rDNA sequences obtained from mouse terminal ileum intestinal samples. (*n* values for Unt = 25; HC = 13; PD = 16). Unt vs. HC, by Kruskal–Wallis test, *p* = 0.982. Unt vs. PD, Kruskal–Wallis test, *p = 0.0335. HC vs. PD, Kruskal–Wallis test, *p = 0.0344). **B** Beta diversity evaluated by Principal Coordinate Analysis (PCoA) based on the Bray–Curtis index of OTUs derived from 16S rDNA sequencing of mouse terminal ileum intestinal samples. (*n* values for Unt = 25; HC = 13; PD = 16). Unt vs. HC, by PERMANOVA test, *p* = 0.3799. Unt vs. PD, PERMANOVA test, *p = 0.0126. HC vs. PD, PERMANOVA test, *p = 0.0126). **C** Pie charts showing the proportional taxonomic composition at the genus level of terminal ileum intestinal samples. **D** Differential abundance of segmented filamentous bacteria (SFB) and (**E**) Differential abundance of *Enterobacteriaceae* in mouse terminal ileum intestinal samples (n values for Unt = 25; HC = 13; PD = 16 analyzed by PERMANOVA with DESeq2 Wald test statistical analysis). Data are presented as mean ± SEM. Statistical significance was calculated using the Kruskal–Wallis test. ∗ *p* ≤ 0.05

To investigate whether PD microbiota transfer (Fig. 1A) could induce changes in motor behavior, we assessed mice’s motor and memory abilities using several behavioral tests. We found that PD microbiota transfer induced motor impairment in the beam walking test (8 mm) (Fig. 1B), increased the Hindlimb clasping score (Fig. 1C) and decreased the latency in the inverted grip test (Fig. 1D). This consistently induced motor behavior alterations were observed both in single donor-recipient and multiple donor-recipient pairs from mice treated with PD microbiome, whereas no such alterations were observed following transplantation from HC donors neither single donor-recipient nor multiple donor-recipient pairs (Figure S3A**-**C). The transfer of PD microbiota also altered mice odor discrimination, consistent with observations in PD patients (Fig. 1E) but did not induce anxiety (Fig. 2D) or altered memory function as assessed by the T-maze test (Figure S2G**, **H). Interestingly, the absence of changes in distance traveled, mean velocity, and resting time suggests that the primary motor capabilities remain relatively intact at this stage of the disease model, or that the test duration and sensitivity may not be sufficient to detect subtle motor deficits. The increased center time may instead reflect a complex interaction between motor, cognitive, and emotional alterations typical of PD (Figure S2C-F). To test whether changes in motor behavior were due to dopaminergic neuronal loss, we analyzed TH levels in the mesencephalon and striatum. Using immunohistochemistry, we observed a decrease in the total number of nigral TH + cells in the SN (Fig. 1F**, **G) and a decrease in TH-positive fibers in the STR (Fig. 1F, H) of PD microbiota-transplanted mice. The loss of dopaminergic neurons also led to a decrease in dopamine levels in the STR (Fig. 1I). Similarly, it was observed that transplants from different PD donors consistently induced the loss of nigral TH + cells in the SN of WT mice, while transplants from HC donors did not produce this effect (Figure S3D, E). There is a lack of consensus on the number of dopaminergic neurons lost in SN or on the levels of dopamine associated with motor impairment, especially in animal models. Our results show that, at the selected time point, there is at least a ~ 30% loss of dopaminergic neurons in the SN occurs and an ~ 52% decrease in striatal dopamine levels, which correlates with the onset of motor symptoms like those observed in human patients upon clinical diagnosis [56].

To confirm that the loss of mesencephalic dopaminergic neurons was associated to the presence of a dysbiotic gut, we first determined the efficacy of gut microbiota colonization from donor fecal material in fecal and ileal mucosa. Mouse fecal pellets were collected at the end of the experiment and the total bacterial DNA extracted was sequenced (Figure S4). Of the 47,843.69 ± 14,872.78 reads obtained per sample, we detected sequences from 115 bacterial genera, 55 families, 33 orders, 14 classes, and 10 phyla. There was a non-statistically significant decrease in alpha-diversity (Figure S4A) and a differential overall community composition (beta-diversity) (Figure S4B) in the fecal material of PD microbiome-transplanted mice (PD mice) compared to HC microbiome-transplanted mice (HC mice) and to untreated animals. We identified specific genera whose relative abundances were altered in the fecal material of PD mice (Figure S4C**-**G), consistent with previous observations in PD patients. We observed a non-statistically significant decrease in *Roseburia* (Figure S4C) and *Butyricicoccus* (Figure S4E). Additionally, Prevotellaceae (Figure S4D) were detected at lower relative abundances in PD mice, whereas *Akkermansia* (Figure S4F) and *Oscillospiraceae* (Figure S4G) were detected at higher abundances compared to HC mice.

### Ileum-associated mucosal dysbiosis disrupts the gut barrier and triggers inflammation

**Fig. 3:**
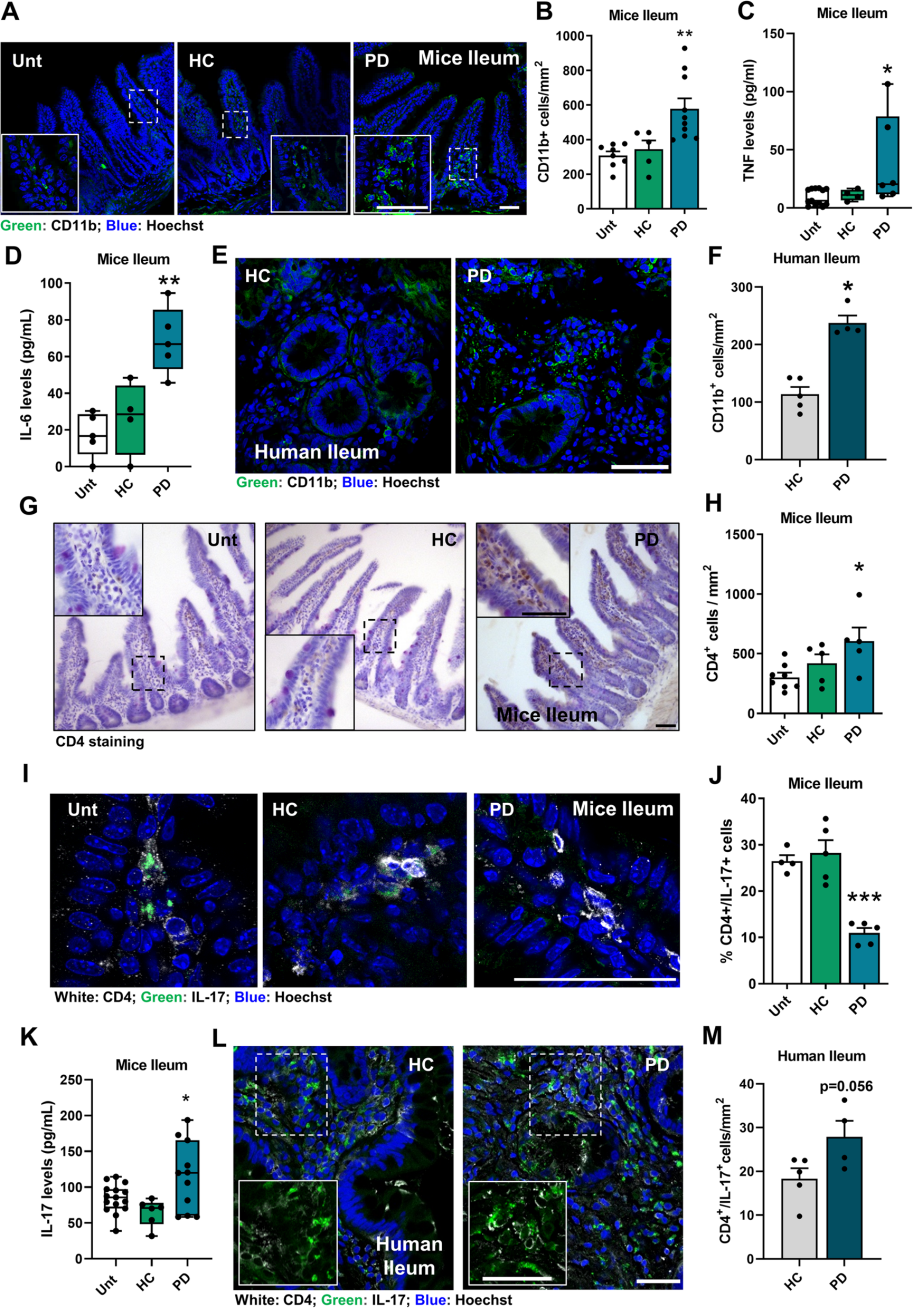
Gut immunity remodeling in PD. **A** Representative immunofluorescence images of transverse mouse ileum sections stained with anti-CD11b. **B** Quantification of CD11b^+^ cells per mm^2^ in the ileum (*n* = 5–9 mice per group). **C**, **D** Measurement of specific inflammatory cytokines by ELISA. **C** TNF (*n* = 4–13 mice per group), (**D**) IL-6 (*n* = 4–5 mice per group). **E** Representative immunofluorescence images of human terminal ileum sections stained with anti-CD11b. **F** Quantification of CD11b^+^ cells per mm^2^ in the ileum (*n* = 4–5). **G** Representative photomicrographs images of transverse ileum sections stained with anti-CD4. **H** Quantification of CD4^+^ cells per mm^2^ in the ileum (*n* = 5–8 mice per group). **I** Representative immunofluorescence images of Th17 cells (CD4^+^/IL17^+^) in transverse sections of the ileum. **J** Quantification of Th17 cells (CD4^+^/IL17^+^) cells per mm^2^ in the ileum (*n* = 4–5 mice per group). **K** IL-17 levels (pg/mL) in the ileum (*n* = 6–15 mice per group) measured by ELISA. **L** Representative images of human terminal ileum sections stained with anti-IL-17 and CD4. **M** Quantification of CD4 + /IL**-**17 + cells per mm.^2^ in human ileum (*n* = 4–5). Data for histological analysis and IL**-**17 determination were obtained from different animal cohorts. *p < 0.05, **p < 0.01, ***p < 0.001, using one-way ANOVA with Dunnet´s test (**B**, **D**, **H** and **J**, **K**) or Kruskal–Wallis with Dunn´s test (**C**) and unpaired Student´s t**-**test (**F** and **M**). Data are expressed as mean ± SEM.. Scale bars are 50 µm. See also Tables S3-S4 and Figures S3

The composition of the fecal microbiota is characterized by a high ratio of transient bacteria and appears to have less influence on gut immune responses than the mucosa-associated microbiota [57]. The focus of this study was on the ileum-associated mucosa due to our previous findings that gut inflammation in mice treated with a bacterial toxin was significantly more robust in the ileum compared to the duodenum or the jejunum [4]. Our findings in PD mice indicate that changes in the microbiota linked to the ileal mucosa following transplantation led to a pro-inflammatory state. On average, there were 13,998.94 ± 15,423.61 reads per ileum sample, and sequences for 97 bacterial genera, 57 families, 37 orders, 13 classes, and 10 phyla were identified. There was a statistically significant increase in the alpha diversity of ileal mucosa-associated microbiota communities between HC and PD mice (Fig. 2A), with a similar overall community composition (beta-diversity; Fig. 2B). Several genera had altered relative abundance in animals colonized with PD microbiota (Fig. 2C). The ileal mucosa of PD mice showed a lower abundance of segmented filamentous bacteria (SFB) (Fig. 2D). Similarly, in the multiple-donor paradigm, it was observed that transplants from different PD donors led an erosion of SFB from ileum mucosa, while no such changes were seen with transplants from HC donors (Figure S3F). Additionally, we observed an increase in the relative abundance of Enterobacteriaceae in PD mice (Fig. 2E), a family that includes several pathogenic bacteria of the genera *Klebsiella*, *Enterobacter*, *Salmonella*, *Escherichia coli*, *Proteus* and others commonly found in the human intestinal tract. Gut dysbiosis in PD mice associates with the erosion of SFB and the increase of Enterobacteriaceae from the ileal mucosa that may correlate with the disruption of intestinal epithelial cell signaling, leading to an increase in CD11b^+^ macrophages or dendritic cells in the ileum lamina propria of PD mice (Fig. 3A**, **B). Moreover, these alterations were also seen when using fecal material from different PD patients. (Figure S3G, H). This increase in CD11b^+^ cells promoted tissue inflammation, resulting in the production of pro-inflammatory mediators such as TNF (Fig. 3C, Figure S3I), IL**-**6 (Fig. 3D), and IL**-**8 (Figure S5A). The levels of CD11b^+^ cells were also significantly increased in human ileal samples from PD patients (Table S3; Fig. 3E**, **F). In this proinflammatory state, we observed an increase in CD4^+^ recruitment to the mouse intestine (Fig. 3G**, **H). Gut microbiota are key players in the differentiation of CD4^+^ cells into Th17 cells [58]. Ileal SFB in mice are known to actively stimulate gut immunity by inducing homeostatic Th17 cells that maintain the integrity of the intestinal barrier [22, 58, 59]. We have demonstrated that the number of tissue-resident homeostatic Th17 cells (CD4 + /IL**-**17 + cells) decreases, while the levels of IL**-**17 in the ileum increase, likely attributed to CD8 + cell activity (Fig. 3I**-**K) [22]. In the multiple-donor PD paradigm, transplants from different PD donors led to an increase in ileal IL**-**17 levels, while no changes were seen with HC donor transplants (Figure S3J). Our findings in mice suggest a specific effect of bacteria in the ileum mucosa, as PD microbiota transfer did not induce changes in IL**-**17 levels in the duodenum, jejunum, or cecum (Figure S5B**-**D). However, we observed a non-statistically significant increase in Th17 cells (CD4^+^/IL**-**17^+^ cells) (*p* = 0.056) in the terminal ileum of PD patients (Fig. 3L**, **M). It is possible that the recruitment rate of CD4^+^ T cells in mice exceeds the differentiation process at our experimental time point due to an initial acute phase of the disease with a different inflammatory profile, potentially characterized by a decrease in homeostatic Th17 cells induced by SFB [59]. The ileum mucosa of both HC individuals and PD patients did not reveal SFB sequences in our preliminary data (Table S4). However, phylogenetically related SFB have been previously detected in humans, and the first human-specific SFB genome has recently been sequenced [60]. In adults, SFB may be replaced by *Bifidobacterium* strains, which are capable of inducing Th17 cells without inflammation, although the activation may occur through a distinct transcriptional program compared to SFB [61]. Our preliminary data in two PD patients terminal ileum samples show a depletion of *Bifidobacterium* bacteria (Table S4). However, the role of SFB and of *Bifidobacterium* bacteria in the differentiation of Th17 cells in PD requires further study.

**Fig. 4:**
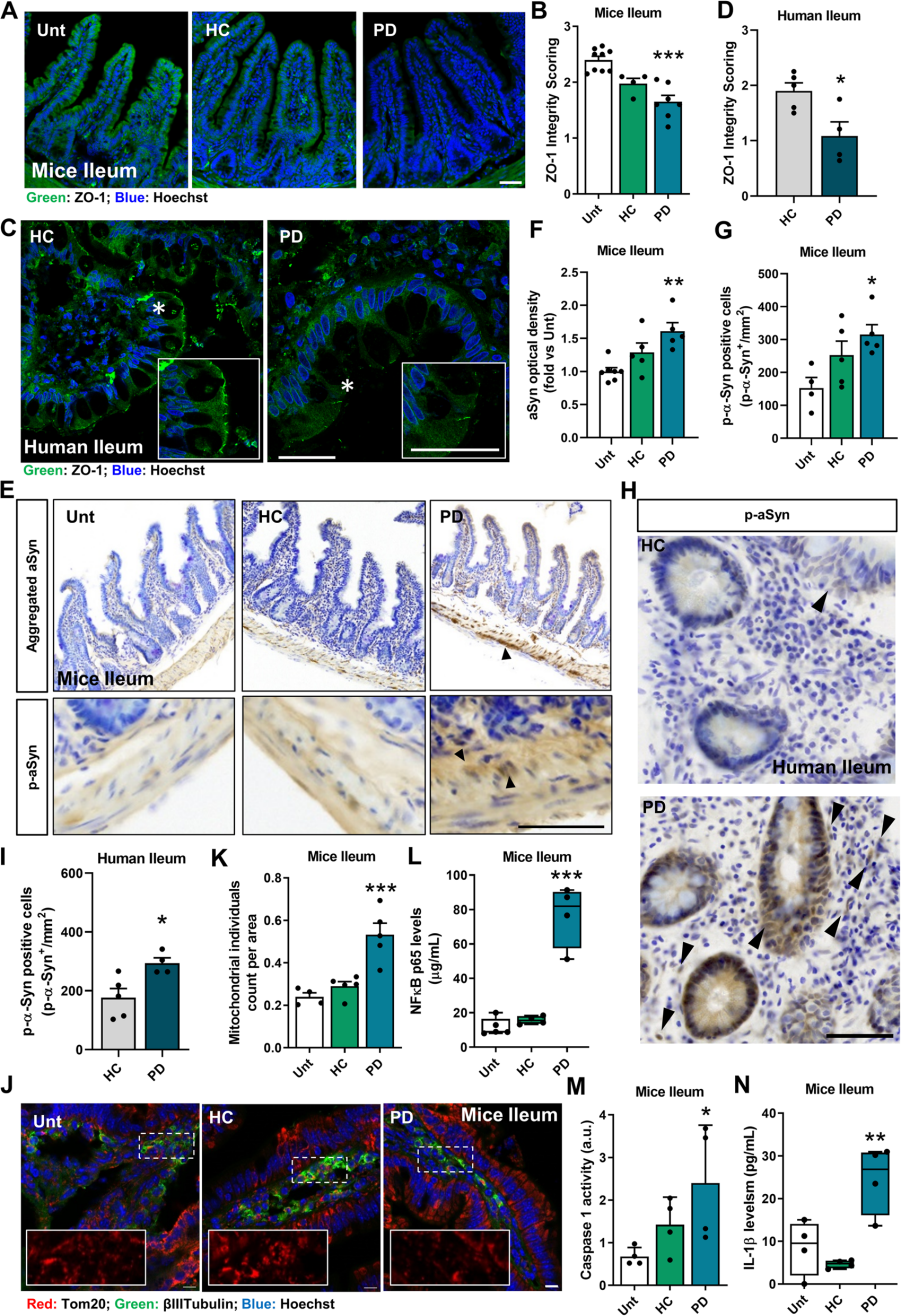
Gut microbiota of PD patients induces mouse ileal inflammation. **A**, **B** Assessment of intestinal barrier integrity. **A** Immunofluorescence staining for zonula occludens**-**1 (ZO**-**1). **B** ZO**-**1 integrity score (*n* = 4–9 mice per group). **C** Representative images of human ileum sections stained with anti-ZO-1. **D** ZO**-**1 integrity score (*n* = 4–5). **E** Photomicrographs showing histology for aSyn aggregates and p-aSyn (pS129) immunoreactivity in the ileum from fecal material-treated mice. **F** Quantitative analysis of OD for aSyn aggregates immunoreactivity in myenteric plexuses. Data were normalized to the untreated group (*n* = 5–7 mice per group). **G** Quantification of p-aSyn positive cells/mm^2^. (*n* = 4–5 mice per group). **H** Photomicrographs represent histology for p-aSyn (S129P) immunoreactivity in the terminal ileum of human subjects. **I** Quantification of p**-**aSyn positive cells/mm^2^ (*n* = 4–5). **J** Representative immunofluorescence images of the mitochondrial network (Tom20) in the myenteric plexus of untreated, HC mice and PD mice. **K** Quantification of mitochondrial individuals in βIII-tubulin-positive neurons per mm.^2^ in the ileum (*n* = 4–5 mice per group). **L-N** Proinflammatory markers measured by ELISA. **L** NFκB (*n* = 4–5 mice per group), (**M**) Caspase**-**1 activity (*n* = 4 mice per group) and (N) IL-1β (*n* = 4 mice per group). *p < 0.05, **p < 0.01, ***p < 0.001, by one-way ANOVA with Dunnet´s test (**B**, **F**, **G**, **K**-**N**) and unpaired Student´s t**-**test (**D** and **I**). Data are mean ± SEM. Scale bars are 50 µm in all images apart from J (10 µm). See also Tables S3 and Figure S3 and S5

Elevated levels of TNF-α, which is also produced by pro-inflammatory Th17 cells [22], are recognized to increase intestinal permeability by internalizing tight junction proteins, specifically ZO**-**1 [62]. We noted a significant reduction in ZO-1 in the ileum of both PD mice (Fig. 4A**, **B; Figure S3K**-**L) and PD patients (Fig. 4C**, **D), indicating a loss of integrity in the intestinal barrier. Indeed, we also saw an increase in calprotectin in the fecal material of our PD mice (Figure S5E). The accumulation of aSyn aggregation, a key feature of PD, is associated with a pro-inflammatory environment and loss of intestinal barrier integrity. In addition, *E. coli* that synthesizes curli protein, which possess amyloidogenic potential, also caused gastrointestinal dysfunction and aggregation of aSyn in both the gut and brain, which is linked to motor impairment [13, 63]. We discovered that mice who received fecal material from individuals with PD exhibited elevated levels of aSyn aggregates and p-aSyn in the ileum (Fig. 4E**-**G, Figure S3M**-**N, Figure S5F**, **G). These findings were consistent with the results observed in humans (Fig. 4H**, **I). The ileal neuronal mitochondrial network is fragmented (Fig. 4J, K), likely due to an accumulation of aSyn, as demonstrated by our group using neuronal cells [4, 64, 65]. Indeed, we observed a positive correlation between fragmented mitochondria in the ileal neurons and cells positive for p-aSyn. (Figure S5H). Bacterial pathogen-associated molecular patterns (PAMPs) and/or mitochondrial damage-associated molecular patterns (DAMPs) can activate innate immunity by inducing NF-kB activation. This, in turn, promotes caspase 1 activation and IL-1β production (Fig. 4L**-**N). An increase in IL**-**10, an anti**-**inflammatory cytokine, was observed (Figure S5I), likely as a compensatory measure to deal with the increased intestinal inflammation.

### Peripheral gut inflammation and blood–brain barrier permeabilization

**Fig. 5:**
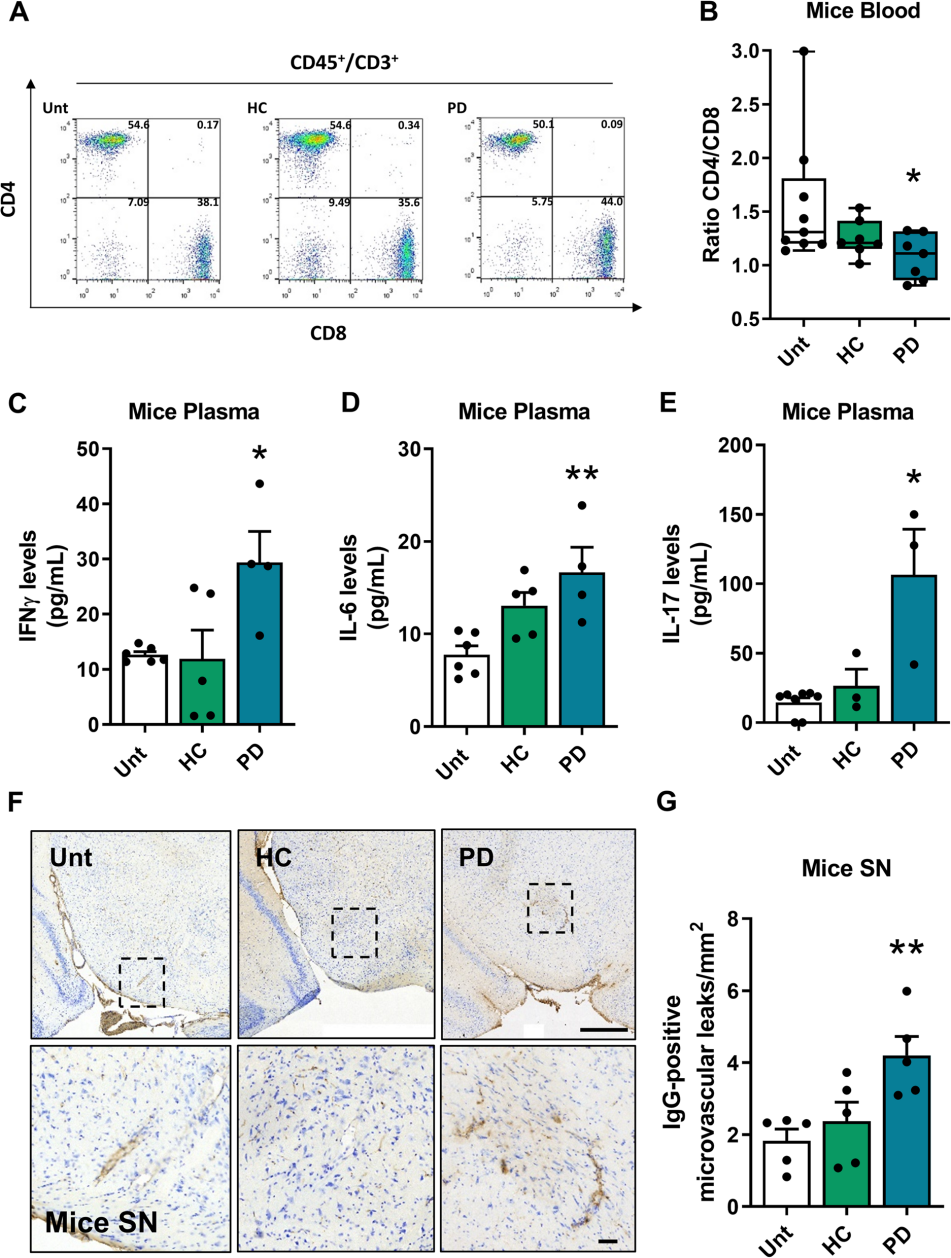
Systemic inflammation and permeabilization of the blood–brain barrier. **A** Representative dot plots of CD45^+^CD3^+^CD4^+^ and CD45^+^CD3^+^CD8^+^ populations in serum samples by flow cytometry. **B** Quantification of the CD4/CD8 ratio (*n* = 7–9 mice per group). **C**-**E** Measurement of specific inflammatory cytokines in mouse plasma by ELISA. (C) IFNγ (*n* = 4–6 mice per group), (**D**) IL-6 levels (*n* = 4–6 mice per group) and (**E**) IL**-**17 levels (*n* = 3–8 mice per group). **F** Representative immunohistological images of SN coronal sections stained with IgG. (G) Quantification of IgG-positive microvascular leakage per mm^2^ in the SN (*n* = 5 mice per group). *p < 0.05, **p < 0.01, using one-way ANOVA with Dunnet´s test (**C**-**E** and **G**) or Kruskal–Wallis with Dunn´s test (**B**). Data are mean ± SEM. Scale bars are 50 µm and 500 µm (upper panel). See also Figure S6

Intestinal inflammation and peripheral recruitment of intestinal CD4^+^ cells result in an imbalance in the distribution of lymphocyte population in the blood. We observed changes in the CD4^+^/CD8^+^ ratio in the blood of PD mice (Fig. 5A**, **B), caused by a decrease in CD4^+^ cells and an increase in CD8^+^ cells (Figure S6A, B). This alteration is linked to a rise in pro-inflammatory cytokines, such as IFNγ and IL-6 in mice plasma (Fig. 5C**, **D) and IL**-**17 in mice and human plasma (Fig. 5E; Figure S3O and Figure S6C), suggesting systemic involvement. Systemic inflammation may result in increased permeability of the BBB [43]. Positive microvascular leakage was observed in the SN of PD mice (Fig. 5F**, **G), while no changes were detected in the STR or cortex (CX) (Figure S6D-G). These findings are noteworthy, as previous studies have shown that the CX and STR exhibit increased permeability to blood-borne components compared to midbrain areas in C57BL/6 mice [66]. It is important to note that this observation is limited to C57BL/6 mice and may not be generalizable to other strains. Additional research is required to establish whether BBB in midbrain regions is more sensitive to inflammation or, the fact that there is a dopaminergic degeneration in course, facilitates this BBB leakage. It is also possible that as PD progresses and affects other upstream areas, the BBB associated with those areas will also be impacted.

### α-Synuclein aggregation in the dorsal motor nucleus of the vagus and substantia nigra in PD mice

**Fig. 6:**
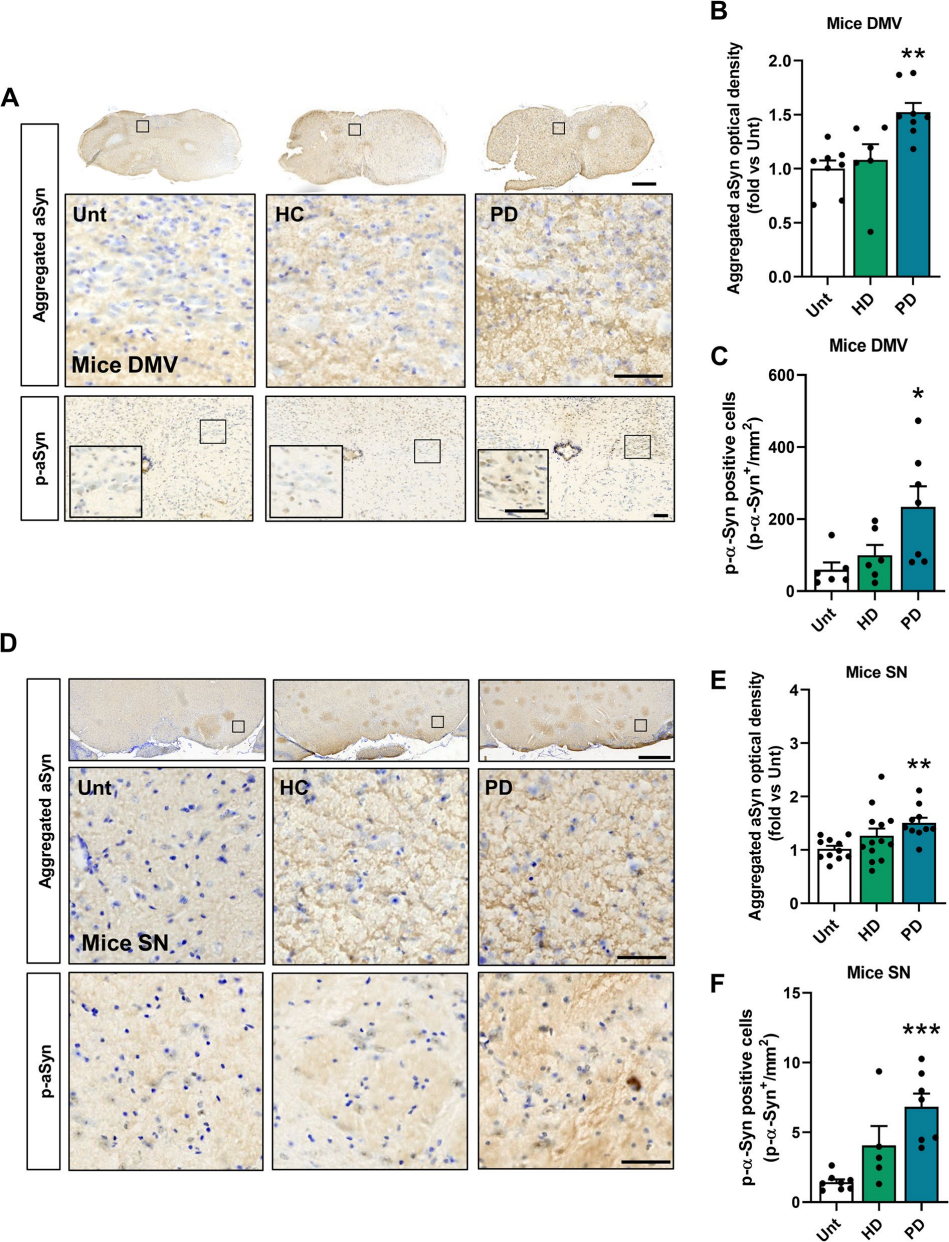
aSyn pathology in the Dorsal Motor Nucleus of the Vagus and Substantia Nigra. **A** aSyn aggregates and p**-**aSyn (pS129) histological immunoreactivity in the dorsal motor nucleus of the vagus (DMV). **B** Quantitative analysis of OD for aSyn aggregates immunoreactivity in the DMV. Data are normalized to the untreated group (*n* = 6–8 mice per group). **C** Quantification of p-aSyn (S129P) positive cells/mm^2^ in DMV (*n* = 6–7 mice per group). **D** Photomicrographs showing histology for aSyn aggregates and p-aSyn (S129P) immunoreactivity in SN from fecal material-treated mice. **E** Quantitative analysis of OD for aSyn aggregates immunoreactivity in SN. Data are normalized to the untreated group (*n* = 10–13 mice per group). **F** Quantification of p**-**aSyn (S129P) positive cells/mm.^2^ immunoreactivity in SN (*n* = 5–8 mice per group). *p < 0.05, **p < 0.01, ***p < 0.001 by one**-**way ANOVA with Dunnet´s test (**B**, **E** and **F**) or Kruskal–Wallis with Dunn´s test (**C**). Data are mean ± SEM. Scale bars are 50 µm (magnified inner square) and 1 mm. See also Figure S7

Aggregates of aSyn in LBs or LNs are significant histopathological features of PD. Recent data from human and mouse models of PD suggest that the upregulation of aSyn expression and subsequent oligomerization initially occur in enteric neurons of the gut. The aSyn then travels from the gut to the brain via the parasympathetic and sympathetic branches of the autonomic nervous system [67]. In our mouse model, CD4^+^ cell infiltration into the brain parenchyma was not observed at the studied time point (Figure S6H). This suggests a possible involvement of the vagus nerve as a possible route of pathology propagation. Pathological aSyn was detected in the dorsal motor nucleus of the vagus (DMV) (Fig. 6A**-**C) and finally in the mesencephalon (Fig. 6D**-**F, Figure S7A**, **B) following to Braak’s staging hypothesis [5, 6]. Accordingly, our previous data does not indicate an increase in aSyn immunoreactivity in the cortex of PD mice (Figure S7C).

**Fig. 7:**
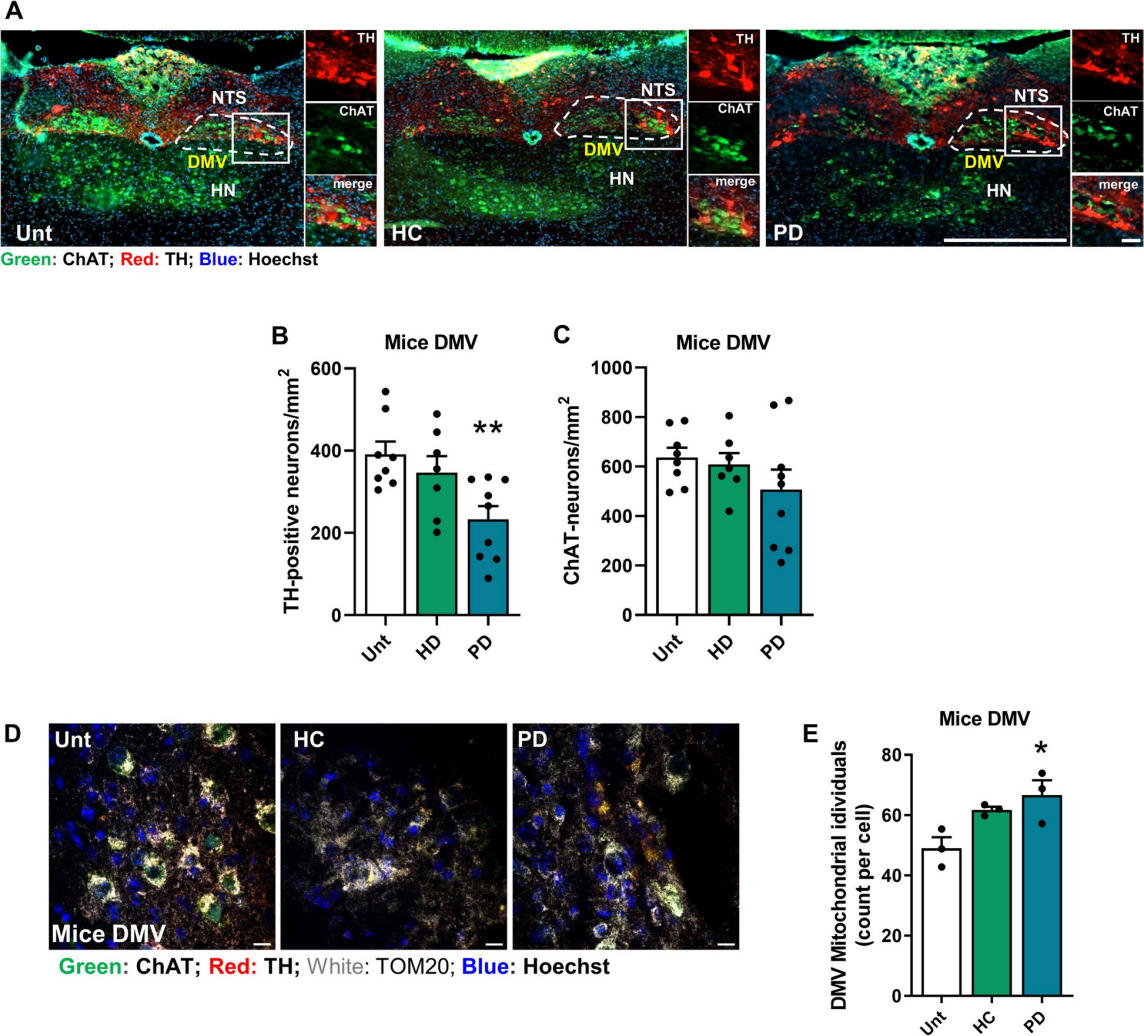
Enhanced sensitivity of DMV TH + neurons. **A** Representative immunofluorescence photomicrographs of the localization of tyrosine hydroxylase (TH) and choline acetyltransferase (ChAT)-positive neurons in the DMV from untreated and HC and PD transplanted mice. **B** Quantification of the number of TH-positive neurons per mm^2^ in the DMV region (*n* = 7–9 mice per group). **C** Quantification of the number of ChAT**-**positive neurons per mm^2^ in the DMV region (*n* = 7–9 mice per group). **D** Representative immunofluorescence photomicrographs of Tom20 in the DMV from untreated and HC and PD transplanted mice. **E** Quantification of the mitochondrial individuals in TH**-**positive neurons per mm.^2^ in the DMV region (*n* = 3 mice per group). *p < 0.05 and **p < 0.01 using ANOVA one**-**way with Dunnet´s test. Data are represented as mean ± SEM. Scale bars are 500 µm except in inner box 50 µm (**A**) and 10 µm (**D**). See also Figure S7

Furthermore, in the DMV, we noted a decrease in the number of TH-positive neurons in PD mice, while the number of ChAT-positive neurons remained unchanged (Fig. 7A**-**C). This suggests that dopaminergic neurons are more vulnerable and may be affected by vagal catecholaminergic fibers located in the abdominal vagus nerve, where approximately 70% of the neurons are TH-positive [68]. Correlational analysis indicates that the amount of phosphorylated aSyn (p**-**aSyn) in the DMV is linked to the depletion of DMV TH^+^ neurons (Figure S7D), but not ChAT ^+^ neurons (Figure S7E). We observed an increase in the number of fragmented mitochondria in the DMV of PD mice (Fig. 7D**, **E). Pearson correlation analysis showed that pathological p-aSyn levels increased mitochondrial fragmentation in DMV (Figure S7F).

### Neuronal innate immunity and neuroinflammation activation in PD mice

**Fig. 8:**
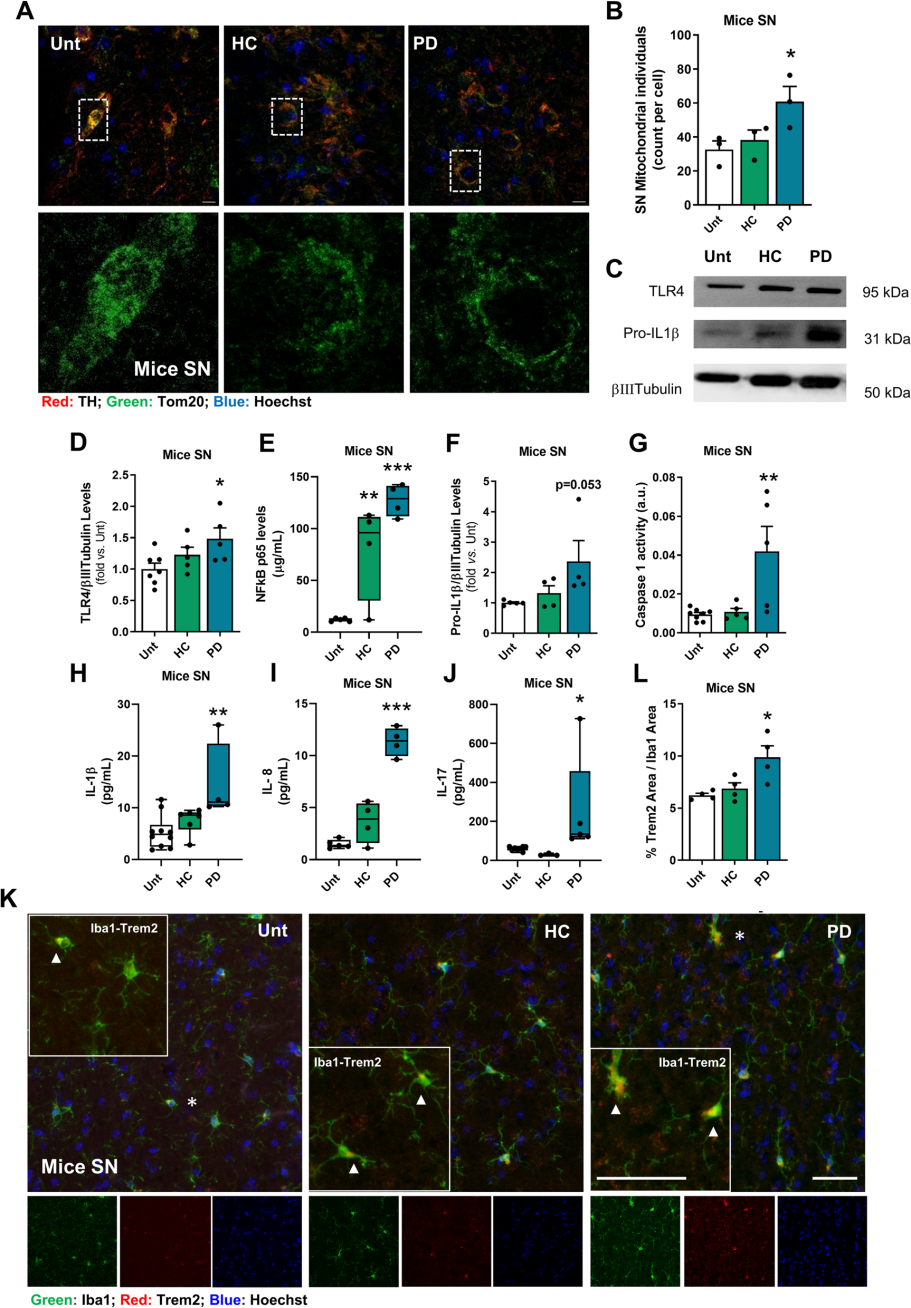
Innate immunity and neuroinflammation activation in the PD mouse model. **A** Representative immunofluorescence micrographs of midbrain coronal sections stained with Tom20 and TH. **B** Quantification of the mitochondrial individuals in TH-positive neurons per mm.^2^ in the SN (*n* = 3 mice per group). **C** Representative immunoblot for TLR4 and pro-IL-1β levels. The blots were reprobed for βIII**-**tubulin to confirm equal protein loading. **D** Densitometric analysis of TLR4 levels normalized to βIII-tubulin (*n* = 5–7 mice per group) measured by WB. **E** NFκB levels in μg/mL (*n* = 4–5 mice per group) measured by ELISA. **F** Densitometric analysis of pro-IL-1β levels normalized to βIII-tubulin (*n* = 4–5 mice per group) measured by WB. **G**-**J** Proinflammatory markers measured by ELISA. **G** Caspase**-**1 activation (*n* = 5–8 mice per group). **H** IL-1β levels in pg/mL (*n* = 4–10 mice per group). **I** IL**-**8 levels in pg/mL (*n* = 4–5 mice per group). **J** IL-17 levels in pg/mL (*n* = 3–8 mice per group). **K** Representative images of coronal brain sections stained with Iba1 (microglial and macrophage-specific calcium**-**binding protein), Trem2 (Triggering Receptor Expressed on Myeloid Cells 2) and Hoechst 33,342 as a nuclear marker in SN. Enlarged boxes show the area of Trem2 included in the Iba1 signal. **L** Percentage of Trem2 area included in Iba1 expression (*n* = 4 mice per group). Data were obtained from different animal cohorts. *p < 0.05, **p < 0.01, ***p < 0.001, by one-way ANOVA with Dunnet´s test (**B**, **D**-**G** and **I**-**L**) or Kruskal–Wallis with Dunn´s test (**H**). Data are mean ± SEM. Scale bars are 10 µm (**A**) and 50 µm (**K**). See also Figure S8

Mitochondrial dysfunction and fragmentation are well-established features of PD [33, 34]. In our PD mouse model, we observed an increase in mitochondrial fragmentation in the substantia nigra (SN) (Fig. 8A**, **B). Pearson correlation analysis also showed that pathological aSyn levels potentiate SN mitochondrial fragmentation (Figure S7G). We isolated mouse mesencephalic mitochondria and observed a reduction in mitochondrial function, specifically a decrease in in ATP synthesis (Figure S8A**-**D), in PD mice. However, cortical mitochondria isolated at the same time point from PD mice were unaffected (Figure S8E**-**H).

Fragmentation of neuronal mitochondria can lead to the exposure of cardiolipin, which is a recognized DAMP [69] that activates innate immune responses in a self-amplified loop culminating in neurodegeneration [64, 70]. In this study, we observed an increased expression of TLR4 in the mesencephalon of PD mice (Fig. 7C**, **D) and activation of NFkB (Fig. 7E), which binds to the promoter region of the IL**-**1β gene in the nucleus and induces its transcription [71]. Activation of Caspase 1, which cleaves pro-IL1β, was observed in PD mice (Fig. 7C, F**-**G), resulting in an increase in mature IL-1β levels (Fig. 7H, Figure S3P). The same activation of this innate immune pathway is also present in human SN samples (Figure S8I-K and Table S5). These results strongly suggest that PD microbiota transfer activates innate immunity in the midbrain. Additionally, midbrain levels of IL**-**8 and IL**-**17 were found to increase in PD mice (Fig. 8I**, **J, Figure S3Q) and in the SN of PD patients (Figure S8L). Recent studies have linked IL**-**17 production in the brain to blood–brain barrier (BBB) leakage and the progression of inflammatory diseases [72]. Neurons can initiate an innate immune response that signals microglia to eliminate dead cells, redundant synapses, protein aggregates, and other factors that may compromise the CNS [73]. Activated microglia with enlarged cell bodies and processes, as visualized by Iba1, were observed (Fig. 8K). Additionally, an increased localization of Trem2**-**positive disease-associated microglia was detected (Fig. 8K**, **L). Activated microglia also release pro-inflammatory cytokines, namely TNF and IL-1β, which may promote further BBB permeabilization and subsequent infiltration of peripheral leukocytes into the CNS [72].

### Sequential pathological progression in PD

**Fig. 9:**
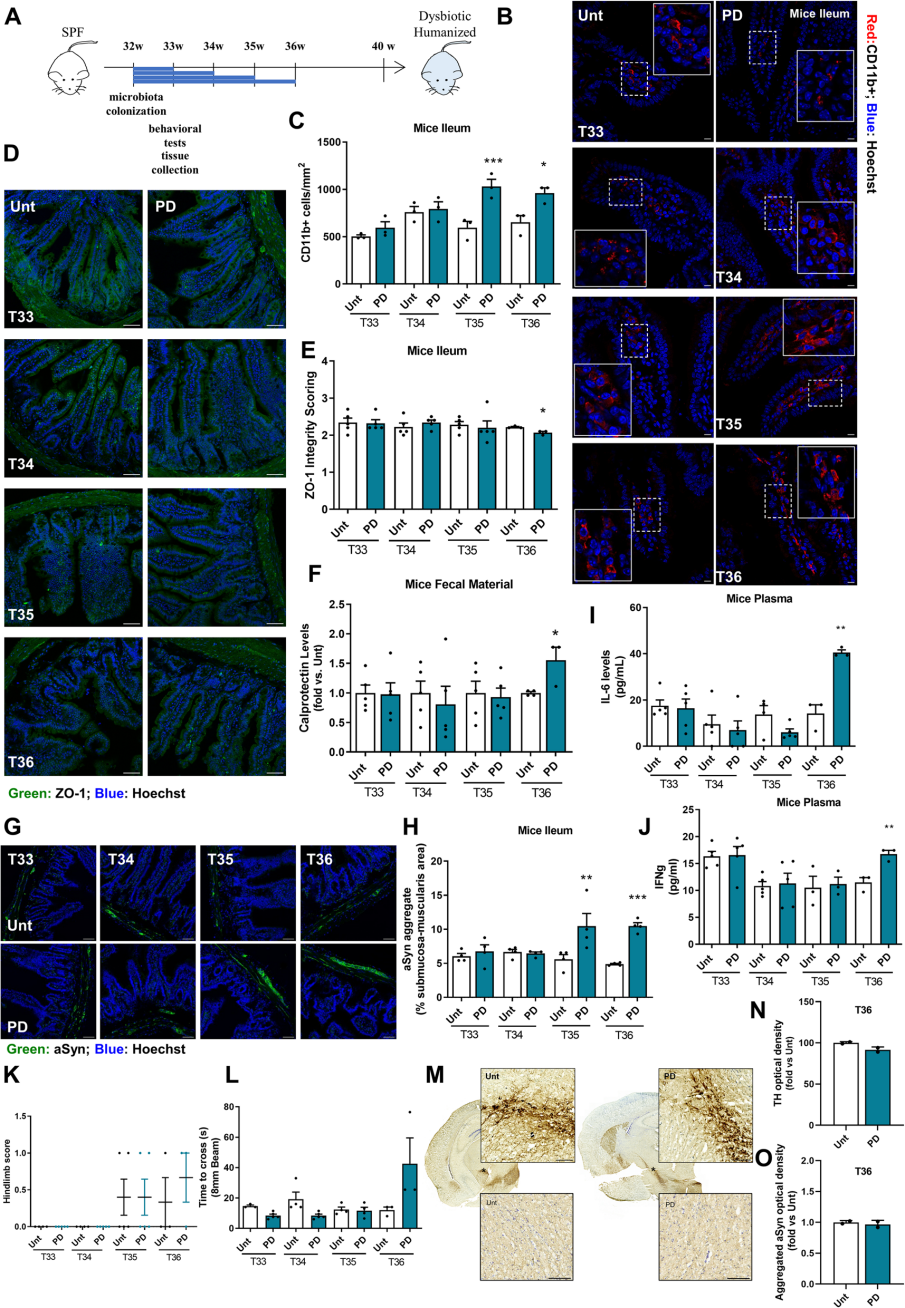
Gut-First PD Progression. **A** Experimental design. **B** Representative immunofluorescence images of transversal mice ileum sections stained with anti**-**CD11b. **C** Quantification of CD11b^+^ cells per mm^2^ in the ileum (*n* = 3 mice per group). **D** Representative images of human ileum sections stained with anti-ZO-1. (E) ZO-1 integrity score (*n* = 3 mice per group). **F** Fecal material calprotectin levels (*n* = 4–5 mice per group) measured by ELISA. **G** Photomicrographs represent histology for aSyn aggregates immunoreactivity in the ileum from fecal material-treated mice. **H** Quantitative analysis of optical density (OD) for aSyn aggregates immunoreactivity in myenteric plexuses. (*n* = 4 mice per group). **I**-**J** Measurement of specific inflammatory cytokines in mice plasma by ELISA. **I** IL-6 and (**J**) IFNγ levels (*n* = 3–5 mice per group). **K** Hindlimb clasping reflex was monitored, as a quick phenotypic neurological scoring system for evaluating disease progression (*n* = 3–5 mice per group). **L** Balance and motor coordination performance was assessed with the beam walking test (*n* = 3–5 mice per group). **M** Representative photomicrographs of brain coronal sections immunostained for TH^+^ in the substantia nigra (SN). **N** OD analysis of the TH-positive fibres in the SN group normalized to the untreated group (*n* = 2 mice per group). **O** Quantification of p**-**aSyn (S129P) positive cells/mm^2^ immunoreactivity in SN (*n* = 2 mice per group). *p < 0.05, **p < 0.01, ***p < 0.001 by one-way ANOVA with Dunnet´s test (**C**, **H**-**I**) or unpaired Student´s t**-**test (**E**–**F**, **J**). Data are mean ± SEM. Scale bars in M are 100 µm and 50 µm in the other images

Using our FMT protocol, where PD patients fecal material are given to WT mice for six weeks (every day in the first week and twice a week for the remaining period) we are forcing mice colonization rather than relying on stable colonization, which is crucial for maintaining gut dysbiosis as a chronic event. We show that gut and blood alterations precede brain pathology (Fig. 9). Our results indicate a gut-first development of PD pathology, since gut inflammation was observed only after three weeks of PD fecal transplant in mice (Fig. 9A**-**C). At the end of week 36 of continuous FMT gavage we observed the loss of intestinal barrier (Fig. 9D**-**F). Gut inflammation triggers ileal aSyn aggregation (Fig. 9G**, **H) after 3 weeks FMT. Interestingly, systemic inflammation (Fig. 9I**, **J) appeared after 4 weeks FMT in the same time point where we observed intestinal barrier leakage. At 36 weeks we do not observe significant alteration in motor behavior (Fig. 9K**, **L) due to the maintenance of TH + dopaminergic neurons in the midbrain (Fig. 9M**, **N). Relevant is to observe that at 36 weeks we still do not have aSyn aggregates in the PD mice midbrain, indicating a caudo-rostral propagation of PD pathology. Our data imply that the triggers of gut-first PD, as elegantly proposed by Brundin’s group [74], need time to allow for the involvement of facilitators and aggravators for the pathology to reach the brain.

## Discussion

Recent research has unveiled connections between gut microbiota and human health, suggesting a role for gut dysbiosis in neurodegenerative diseases [75, 76]. PD, with its multifactorial etiology with different triggers, facilitators, and aggravators [74], can be categorized into brain-first and body-first types [77, 78]. Our recent findings showed that a microbial toxin can specifically target the ileal-associated microbiota that regulate Th17 immunity, leading to multiple downstream pathological effects compatible with body-first PD. This toxin also targets neuronal mitochondria inducing its dysfunction, which is also linked to PD neurodegenerative pathway [4]. We hypothesized PD dysbiome may initiate a gut-to-brain toxic pathway leading to PD. In this scenario, the gut dysbiome could serve as an environmental source of unidentified toxins capable of disrupting the protective ileal microbiome and activating known pathways linked to PD [79, 80].

While prior research has shown that transplanting gut microbiota from PD patients accelerates pathology in genetic mouse models [12], the impact on WT mice has not been investigated. Here we show that the transference of gut microbiota from PD patients into WT mice induces PD-like behavior, PD histopathological features and dopaminergic degeneration. The extent of dopaminergic neuron loss in the SN and levels of dopamine in the STR suggest that recipient mice reproduce PD pathology [56]. The fecal microbiota of recipient mice exhibited notable shifts towards pro-inflammatory bacteria and a reduction in anti-inflammatory bacteria, mirroring findings in PD patients [81, 82]. It has been reported that colonization with *Proteus mirabilis* caused dopaminergic neuronal damage, neuroinflammation, and motor deficits in WT mice [14]. Indeed, we also observed an increase in the relative abundance of *Enterobacteriacea*, that contains the *Proteus* genera, in ileal mucosa-associated microbiome. A more detailed species-level analysis is needed to identify specific bacteria responsible for the observed effects and to guide further experimental investigations. However, this dysbiosis, characterized by a decrease in key commensal bacteria with immunomodulatory properties, may disrupt intestinal homeostasis [83], and potentially predict early PD progression [84]. On the other hand, SFB regulate the differentiation of CD4^+^ lymphocytes into Th17 homeostatic cells in the gut mucosa of mice [85]. Interestingly, it was recently described in humans that *Bifidobacterium* in gut-associated mucosa may be involved in the regulation of Th17 response [61]. Further studies are required to investigate the role of *Bifidobacterium* in the differentiation of Th17 cells in PD. The ileum erosion of SFB and the increase in Enterobacteriacea in mice can be correlated to the shift from homeostatic Th17 to an inflammatory Th17 cell phenotype [22], thus perpetuating the chronic inflammatory process. This shift may involve the expression of different ROR transcription factors [86], that are associated with an increase in TNF levels. Additionally, chronic inflammation in human PD patients might lead to sustained IL-17 production by Th17 cells that share Th1 features [86]. While gut Th17-deficient mice are more susceptible to gut infections [87], Th17 and IL-17 in the blood are associated to neuroinflammation and neurodegeneration in PD [88]. Interestingly, an increase of IL-17 was observed in plasma in both mice and PD patients [89, 90]. Dysregulated IL-17 responses can promote inflammation during infection and autoimmunity can also be involved in the initial progression of PD [91]. This pro-inflammatory milieu in the gut may impair intestinal barrier function, evidenced by reduced tight junction expression and calprotectin in the fecal material. This can enable the translocation of harmful substances or pathobionts into the gut mucosa [62, 92]. Consequently, increased pro-inflammatory bacteria like *Oscillospiraceae* in PD mice and elevated TNF-α levels contribute to ZO-1 loss in the ileum, enhancing gut permeability [82].

Pathological aggregates of aSyn are a hallmark of PD progression. Our study revealed aggregated and phosphorylated aSyn in ileum samples from PD-transplanted mice. While retrograde spread of aSyn aggregates from the ENS to CNS is widely theorized [10, 11], its role remains controversial [93, 94]. Some studies reported no differences in colonic aSyn accumulation between PD and HC [95–97]. However, we have found differences in pathological p-aSyn between PD and HC in terminal ileum human biopsies.

It is noteworthy that PD mice exhibit systemic dissemination of gut inflammation, as evidenced by elevated pro-inflammatory cytokine levels and decreased CD4 + lymphocytes in the blood. This mirrors observations of reduced CD4 + T cells in PD patients, implicating a peripheral inflammatory response in PD pathophysiology [2, 98]. Various proinflammatory cytokines can prompt BBB disruption [43, 99], and so the increase in mesencephalic IgG levels suggests that gut and systemic inflammation induced by the PD microbiota may enhance BBB permeability. Interestingly, at this time point, this effect is localized in the SN, with no changes in the striatum or cortex. However, we do not see the recruitment of CD4^+^ T cells to brain parenchyma, which may indicate that dopaminergic degeneration may precede a more compromised BBB. We show that the caudo-rostral progression of aSyn plays a pivotal role in pathology development. Our findings reveal accumulative pathological aSyn along DMV and midbrain but not in STR or cortex. Moreover, the levels of p-aSyn gradually decrease from the gut to the SN. Additionally, we demonstrate that p-aSyn correlates with mitochondrial dysfunction in the ileum, DMV and SN. Certainly, our work suggests that mitochondrial network impairment and fragmentation may result from aSyn accumulation. The correlation between an increase in p-aSyn with increased mitochondrial fragmentation likely initiates a caudo-rostral progression, activating neuronal innate immunity, which in turn signals microglia, inducing neuroinflammation and subsequent dopaminergic neuronal loss [100]. Recent studies indicates that mitochondrial lipid cardiolipin can prompt rapid oligomerization of pathological aSyn inducing mitochondrial dysfunction and cell death [101]. Other authors have reported that bacterial infection could induce mitochondria-specific autoimmune response that ultimately led to PD [102, 103].

## Conclusions

In summary, the dysbiome observed in PD, potentially generating toxin-like compounds, establishes a detrimental gut-to-brain pathway. Immune changes initially detected in both mice and PD patients' gut emphasize the importance of this study, indicating potential novel peripheral biomarkers and strategies to slow PD progression. The aggregation of aSyn in the terminal ileum linked with peripheral inflammation could serve as a prodromal PD biomarker. Integrating intestinal anti-inflammatory drugs into clinical trial frameworks might mitigate intestinal pathology and forestall the cerebral phase of PD.

## Supplementary Information

Supplementary material 1.

## Data Availability

The data that support the findings of this study are available from the corresponding author upon reasonable request.

## Abbreviations

BBB: Blood–brain barrier
CNS: Central Nervous System
ChAT: Choline acetyltransferase
DMV: Dorsal motor nucleus of the vagus nerve
ENS: Enteric Nervous System
GI: Gastrointestinal
HC: Healthy controls
IBD: Inflammatory bowel disease
LBs: Lewy bodies
M.O.M: Mouse on Mouse Blocking Reagent
OD: Optical density
PD: Parkinson’s disease
PBMCs: Peripheral blood mononuclear cells
SFB: Segmented Filamentous Bacteria
STR: Striatum
SN: Substantia Nigra
Th: T helper
TH: Tyrosine hydroxylase
WT: Wild**-**type
p**-**aSyn: Phosphorylated**-**α**-**Synuclein
aSyn: α**-**Synuclein
