# Supplementary material for: Gut-first Parkinson’s disease is encoded by gut dysbiome
Source: Mol Neurodegener. 2024 Oct 24;19:78. doi: 10.1186/s13024-024-00766-0 (PMC11515425; doi:10.1186/s13024-024-00766-0)
Supplement: Supplementary file 1 — Supplementary material 1. [file 13024_2024_766_MOESM1_ESM.pdf]

Supplemental material for:

"Gut-first Parkinson's disease is encoded by gut dysbiome"

Table of Contents

Key Resources Table

Supplemental Tables (S1-S5) and Figures and Figure legends (S1-S8)

Whole representative WB membranes

Key Resources Table

REAGENT or RESOURCE	SOURCE
**Antibodies – IHC/IF**	
Rabbit anti-α-synuclein aggregate antibody [MJFR-14-6-4-2]	Abcam (Cat. No. ab209538)
Mouse anti-phosphorylated α-synuclein biotin-conjugated (pSyn#64) (S129P)	Fujifilm Wako Pure Chemical Corporation (Cat. No. 010-26481)
Rabbit anti-ZO-1	Abcam (Cat. No. ab96587)
Mouse anti-βIIITubulin	Cell Signalling (Cat. No. 4466S)
Rabbit anti-TOM20	Santa Cruz Biotechnology (Cat. No. SC-11415)
Rabbit anti-CD4	Cell Signalling (#25229)
Mouse anti-CD11b	BioRad (MCA711GT)
Rat anti-CD11b	Biorad (MCA711G)
Rabbit anti-tyrosine hydroxylase (TH)	Millipore (Cat. No. AB152)
Rabbit anti-Iba1	FUJIFILM Wako Chemicals (Cat. No. 019-19741)
Sheep anti-Trem2	R&D Systems (Cat. No. AF1729)
Mouse anti-ChAT	Invitrogen (Cat. No. MA5-31383)
Donkey anti-Rabbit IgG H&L Alexa Fluor 488	Abcam (Cat. No. ab150073)
Anti-IL-17 Alexa Fluor 488	Santa Cruz Biotechnology (Cat. No. SC-374218)
Biotinylated anti-rabbit IgG	Vector Labs (Cat. No. BA-1000)

Biotinylated anti-mouse IgG	Vector Labs (Cat. No. BA-9200)
Donkey anti-sheep IgG H&L Alexa Fluor 647	Abcam (Cat. No. ab150179)
Donkey anti-sheep Alexa Fluor 568	Invitrogen (Cat. No A-21099)
Goat anti-mouse Alexa Fluor 488	Molecular Probes, Life Technologies (Cat. No. A11001)
Goat anti-mouse Alexa Fluor 594	Molecular Probes, Life Technologies (Cat. No. A11005)
Goat anti-rabbit Alexa Fluor 488	Molecular Probes, Life Technologies (Cat. No. A11008)
Goat anti-rabbit Alexa Fluor 594	Molecular Probes, Life Technologies (Cat. No. A11005)
Goat anti-chicken Alexa Fluor 594	Molecular Probes, Life Technologies (Cat. No. ab96948)
**Antibodies – Flow Cytometry**	
Mouse anti-CD45 PerCP (Clone 30F11)	Miltenyi Biotec (Cat. No. 130-102-469)
Mouse anti-CD3 FITC (Clone REA641)	Miltenyi Biotec (Cat. No. 130-119-798)
Mouse anti-CD4 APC (Clone REA604)	Miltenyi Biotec (Cat. No. 130-116-487)
Mouse anti-CD8a PE (Clone REA601)	Miltenyi Biotec (Cat. No. 130-123-781)
Rat anti-IgG2a PerCP	Miltenyi Biotec (Cat. No. 130-103-094)

REA Control-FITC	Miltenyi Biotec (Cat. No. 130-113-449)
REA Control-PE	Miltenyi Biotec (Cat. No. 130-113-450)
REA Control-APC	Miltenyi Biotec (Cat. No. 130-113-446)
**Antibodies – Western Blotting**	
Mouse anti-β-actin	SIGMA (Cat. No. A5441)
Rabbit anti-αSynuclein	Cell Signalling Technology (Cat. No. 2642)
Goat anti-rabbit IgG	GE Healthcare (Cat. No. NIF1317)
Goat anti-mouse IgG	Thermo Fisher Scientific (Cat. No. 31320)
Rabbit IL-1β	Santa Cruz Biotechnology (Cat. No. sc-7884)
Mouse anti-TLR4	Santa Cruz Biotechnology (Cat. No. sc-293072)
**Kits**	
NFκB p65 Total SimpleStep ELISA Kit	Abcam (Cat. No. ab176648)
ELISA Kit for Dopamine	MyBioSource (Cat. No. MBS2700357)
Mouse IL-8 ELISA Kit	MyBioSource (Cat. No. MBS776466)
Mouse IFNγ ELISA Kit	R&D Systems (Cat. No. MIF00)

Mouse IL-6 ELISA Kit	R&D Systems (Cat. No. M6000B)
Mouse IL-1β Quantikine ELISA	R&D Systems (Cat. No. MLB00C)
Mouse IL-10 Quantikine ELISA	R&D Systems (Cat. No. PM1000B)
Mouse IL-17 Quantikine ELISA	R&D Systems (Cat. No. DY421-05)
NZY Soil gDNA Isolation kit	Nzytech, Lda (Cat. No. MB21802
Mouse TNF-α Quantikine ELISA	R&D Systems (Cat. No. MTA00B)
Mouse S100A8/S100A9 (Calprotectin) ELISA Kit	Invitrogen (Cat. No. EM67RBX10)
**Chemicals**	
Adenosine 5' diphosphate (ADP) potassium salt	Sigma (Cat. No. A5285)
Antimycin A	Sigma (Cat. No. A8674)
Carbonyl cyanide-4-(trifluoromethoxy)phenylhydrazone (FCCP)	Sigma (Cat. No. C2920)
Carbonyl cyanide m-chlorophenyl hydrazone (CCCP)	Sigma (Cat. No. C2759)
Caspase 1 substrate	Sigma (Cat. No. SCP0066)
Oligomycin	Sigma (Cat. No. J60211)
Polyethyleneimine (PEI)	Sigma (Cat. No. 408700)
Rotenone	Sigma (Cat. No. R8875)
Succinic acid	Sigma (Cat. No. S3674)
Hoechst	Invitrogen (Cat. No. H1399)
Light (0% sugar), fruits of the forest flavored gelatin	Royal®

Banana flavor	LorAnn Oils (Cat. No. 3510-0500)
Almond flavor	LorAnn Oils (Cat. No. 3500-0500)
Histopaque 1083	Sigma (Cat. No. 10831-100mL)
DPX Mountant	Sigma (Cat. No. 06522-100mL)
Vectastain Elite ABC Perox standard kit	Vector Labs. (VCPK-6100)
Normal Goat Serum	Abbkine (Cat. No. BMS0050)
Normal Donkey Serum	Abbkine (Cat. No. BMS0140)
OCT mounting medium	Carl Roth (Cat. No. KMA-0100-51A)
MOM blocking reagent	Vector Labs (Cat. No. MKB-2213-1)

Supplementary Table S1. Demographics and clinical information of PD patients and HC individuals

	Age	Clinical diagnosis	UPDRS	HOHEN & YAHR	MMSE	Constipation duration (years)
**PD**	79	2013	43	2	27	4
**PD**	67	2007	10	2	24-30	16
**PD**	80	2013	38	3	24-30	10
**PD**	72	2016	14	2	24-30	8
**PD**	77	2011	32	2.5	24-30	10
**PD**	73	2015	33	2	27	45
**PD**	76	2013	13	1	28	7
**PD**	67	2009	11	1	29	8
**PD**	84	2002	30	2	25	5
**PD**	57	2010	35	2	30	7
**PD**	71	2016	24	2	30	50
**PD**	71	2010	22	1.5	29	60
**PD**	76	1997	27	2.5	29	5
**PD**	72	2012	25	1.5	29	50
**PD**	76	2015	31	1.5	27	20
**PD**	73	2015	27	2.5	26	2
**PD**	69	2013	18	2	30	6
**PD**	73	2005	28	2	30	20
**PD**	76	2015	17	2	26	10
**PD**	77	2008	44	2	24	4
**PD**	76	2012	40	2.5	25	40
**PD**	66	2011	20	2	30	50
**PD**	69	2011	20	2	30	1
**PD**	80	2013	51	2.5	26	50
**PD**	76	2015	25	1.5	29	2
**PD**	86	2014	31	2	24	20
**PD**	75	2009	38	2	24	30
**PD**	73	2006	28	2	24	20
**PD**	76	2014	14	1.5	25	50
**PD**	81	2014	35	3	24	5
**PD**	80	2010	27	1.5	25	30
**PD**	69	2014	34	2	29	1
**PD**	71	2012	30	1	29	1
**PD**	72	2013	29	2	25	50
**PD**	68	2010	40	2	29	1
**PD**	72	2009	30	2	25	1
**PD**	65	2013	10	2	30	3
**PD**	72	2013	31	2	27	1
**PD**	56	2014	14	2	30	1
**PD**	81	2014	23	1	27	10
**PD**	65	2007	18	2	29	2
**PD**	76	2014	28	2	26	50
**PD**	68	2011	17	1	30	50
**PD**	69	1996	42	2	28	50

**HC**	75	n/a	n/a	n/a	25	none
**HC**	74	n/a	n/a	n/a	27	none
**HC**	55	n/a	n/a	n/a	26	none
**HC**	52	n/a	n/a	n/a	30	none
**HC**	64	n/a	n/a	n/a	30	55
**HC**	63	n/a	n/a	n/a	30	none
**HC**	72	n/a	n/a	n/a	29	none
**HC**	69	n/a	n/a	n/a	29	none
**HC**	71	n/a	n/a	n/a	28	none
**HC**	73	n/a	n/a	n/a	30	50
**HC**	63	n/a	n/a	n/a	28	none
**HC**	78	n/a	n/a	n/a	27	none
**HC**	62	n/a	n/a	n/a	ND	30
**HC**	64	n/a	n/a	n/a	26	4
**HC**	55	n/a	n/a	n/a	30	none
**HC**	57	n/a	n/a	n/a	29	30
**HC**	55	n/a	n/a	n/a	29	30
**HC**	65	n/a	n/a	n/a	29	none
**HC**	73	n/a	n/a	n/a	24-30	none
**HC**	72	n/a	n/a	n/a	24-30	none
**HC**	71	n/a	n/a	n/a	24-30	none

UPDRS = unified Parkinson's disease rating scale (0-147); HY stage = Hoehn and Yahr scale (1: Unilateral with tremor, rigidity and akinesia; 2: Bilateral disease, without balance impairment; 3: Bilateral disease, with balance impairment; 4: Bilateral disease, with cognitive decline and 5: Bilateral disease, with dementia). MMSE = Mini–Mental State Examination (24-30: no cognitive impairment; 20-24: Mild dementia; 13-20: moderate dementia and less than 12: severe dementia). n/a: not applicable. ND: not determined. (Information related to Supplementary Figure S1)

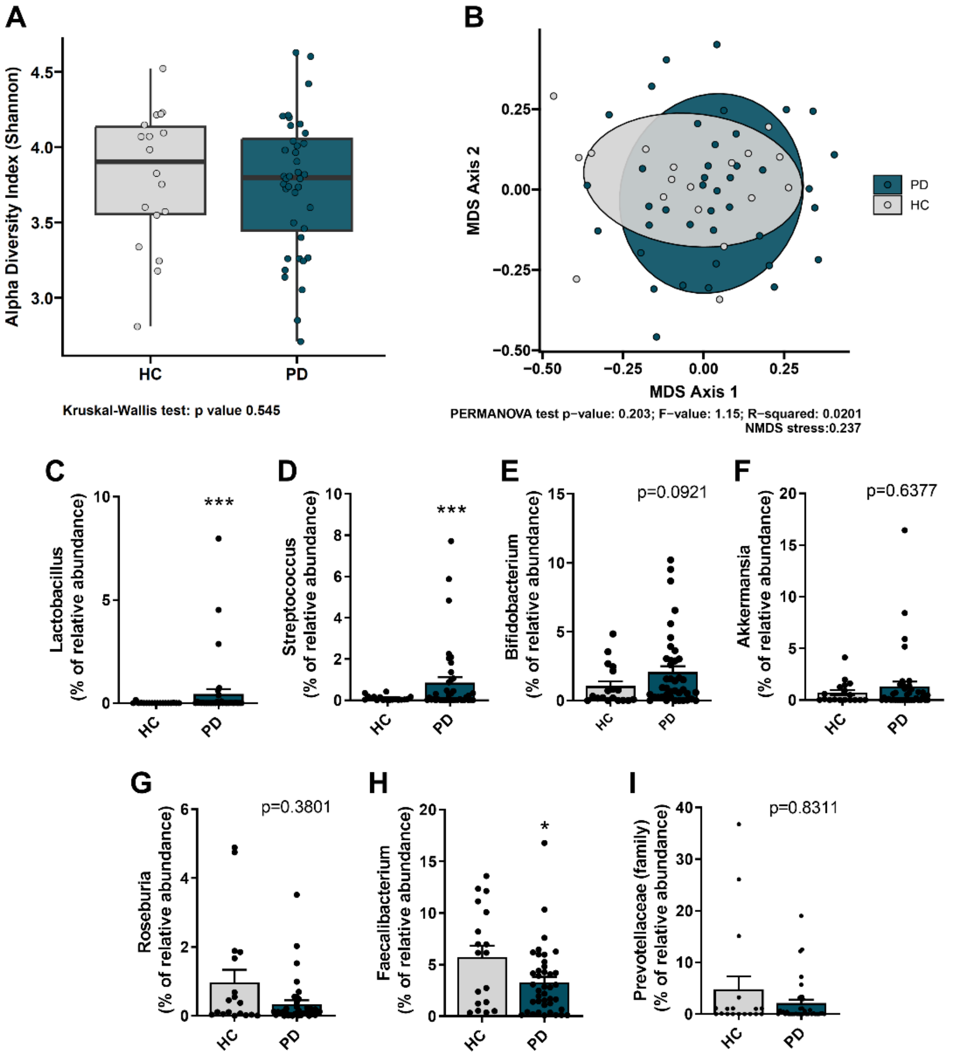

Supplementary Figure S1. PD patients' fecal microbiomes. (A) Alpha diversity was measured using Shannon index at the OTU level in human fecal samples (n values for HC = 18; PD = 40). Comparison between HC and PD conducted using the Kruskal-Wallis test (p = 0.545). **(B)** Beta diversity evaluated using Principal Coordinate Analysis (PCoA) based on the Bray-Curtis index of OTUs derived from 16S rDNA sequencing of human fecal samples (n values for HC = 18; PD = 40). The comparison between HC and PD was conducted using the PERMANOVA test (p = 0.203). **(C-I)** Differential abundance of selected genera in fecal samples from HC and PD patients (n = 18 HC and 40 PD). Statistical analyses were conducted using PERMANOVA with DESeq2 Wald test or Kolmogorov-Smirnov test. Relative abundances of **(C)** *Lactobacillus* (***padj = 5.63E-05); **(D)** *Streptococus* (***padj = 0.000416116); **(E)** *Bifidobacterium* (p = 0.0921). (**F**) *Akkermansia* (p = 0.6377); **(G)** *Roseburia* (p = 0.3801); **(H)** *Faecalbacterium* (*p = 0.0337); (**I**) Prevotellaceae (p = 0.8311). The data is presented as mean ± SEM. Statistical

significance was considered at a level of $*p \leq 0.05$. (Information related to Supplementary Table S1)

Supplementary Table S2. Demographics and clinical information of the selected PD patients and healthy controls whose fecal material was used for transplantation

	**Age**	**Clinical diagnosis**	**UPDRS**	**HOHEN & YAHR**	**MMSE**	**Constipation duration**
**PD1**	79	2013	43	2	NCI	4 years
**HC1**	78	n/a	n/a	n/a	NCI	none
**PD2**	72	2016	14	2	NCI	8
**HC2**	73	n/a	n/a	n/a	NCI	none
**PD3**	67	2007	10	2	NCI	16
**HC3**	72	n/a	n/a	n/a	NCI	none
**PD4**	80	2013	38	3	NCI	10
**HC4**	71	n/a	n/a	n/a	NCI	none
**PD5**	77	2011	32	2.5	NCI	10

UPDRS = Unified Parkinson's Disease Rating Scale (0-147); HY stage = Hoehn and Yahr scale (2: Bilateral disease. without impairment of balance). MMSE = Mini–Mental State Examination (24-30: NCI, no cognitive impairment). (Information related to Supplementary Figure S2 and Figure 1)

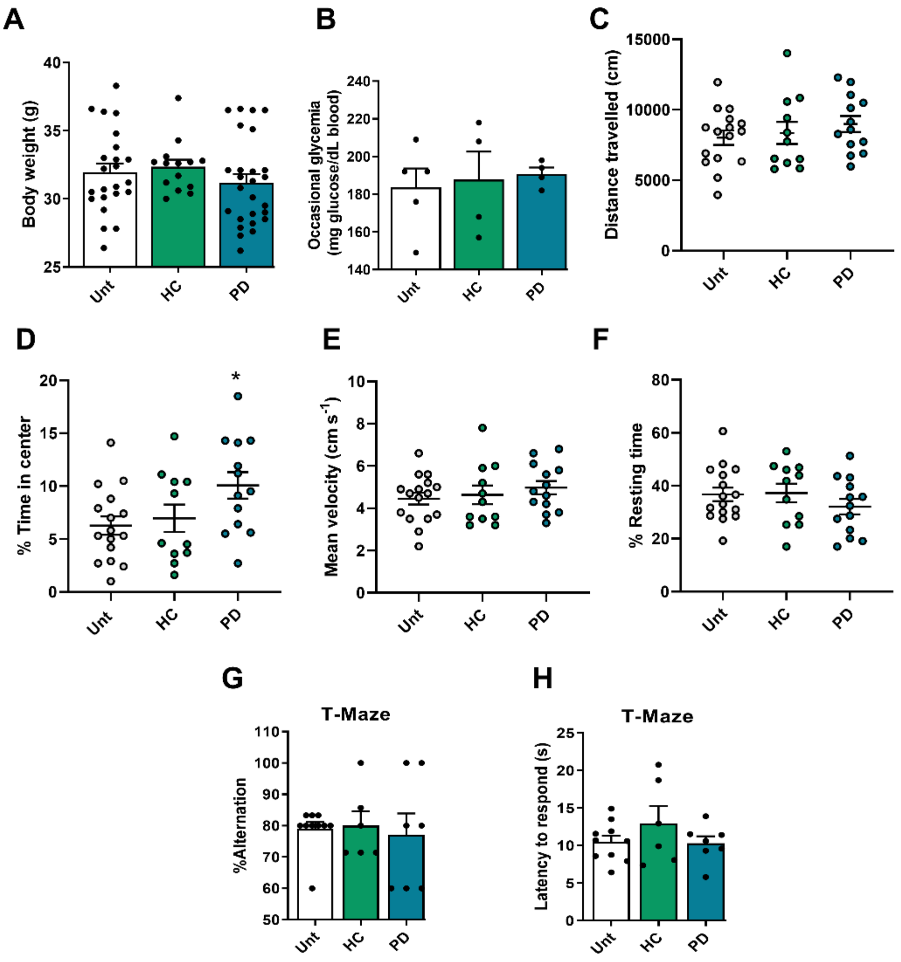

Supplementary Figure S2. Experimental design, mice body weight, glycemia, and behavior.

(A) The body weights of the mice were measured twice a week during the treatment (n = 14-24 mice per group). **(B)** Blood glucose levels were measured at the end of the treatment, and occasional glycemia was calculated (n = 4-6 mice per group). **(C-F)** Locomotor activity was evaluated in an open field arena. (n= 10-19 mice per group). **(C)** Distance traveled (cm), **(D)** % Time spent at the center of the arena, **(E)** mean velocity ($cm.s^{-1}$) and **(F)** % resting time. (**G-H**) Cognitive and memory abilities were assessed using a T-maze. (**G**) The percentage of alternation between arms and (**H**) the latency to respond (s) were measured (n = 5-10 mice per group). *$p < 0.05$, using one-way ANOVA with Dunnet´s test (B-C, E and H) or Kruskal-Wallis with Dunn´s test (A, D, F and G) The data represents the mean ± SEM. (Information related to Figure 1)

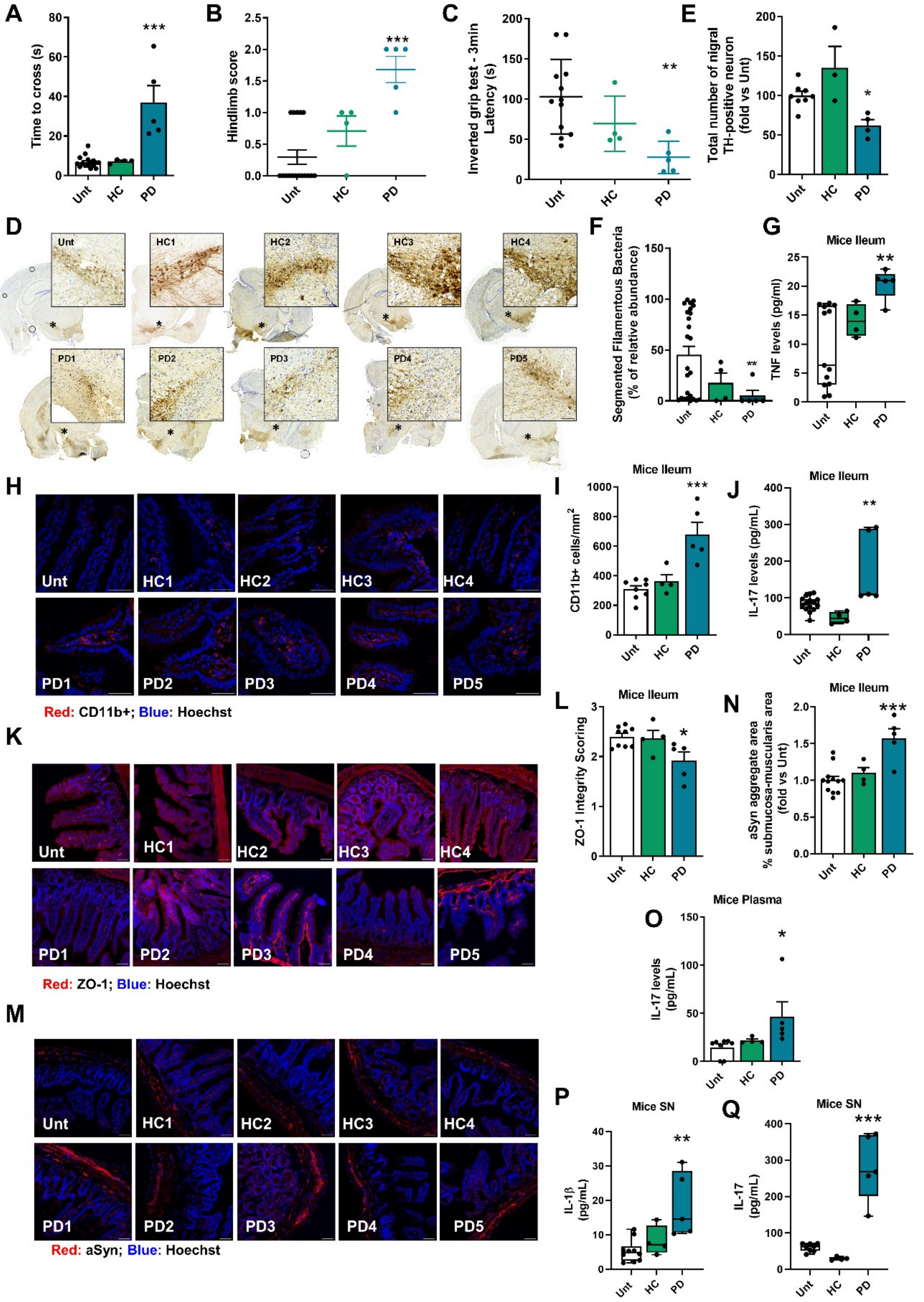

Supplementary Figure S3. Effect of gut microbiome transplantation to WT mice from multiple donor-recipient pairs.

(A) Quantification of the time to cross motor performance test (n = 4-16 mice per group). **(B)** Hindlimb score test performance (n = 4-17 mice per group). **(C)** Inverted grip latency

to fall test (n = 4-12 mice per group). **(D)** Representative immunohistochemistry images of coronal brain sections stained for TH. Magnified images of the substantia nigra (SN), marked with an asterisk, are displayed within the squares. **(E)** Quantification of nigral TH+ neurons presented as fold change versus untreated (n = 3-8 mice per group). **(F)** Quantification of Segmented Filamentous bacteria as percentage of relative abundance (n = 4-25 mice per group) in ileal mucosa. Unt group was used as pre-transplantation gut microbiome control. **(G)** TNF levels (pg/mL) (n = 4-14 mice per group). **(H)** Representative immunofluorescence images of transverse mouse ileum sections stained with anti-CD11b. **(I)** Quantification of CD11b+ cells per mm^2 in the ileum (n = 4-8 mice per group). **(J)** IL-17 levels (pg/mL) (n = 4-14 mice per group). **(K)** Representative immunofluorescence images of transverse mouse ileum sections stained with anti-ZO-1 tight junction protein. **(L)** Quantification of ZO-1 by integrity score assessment (n = 4-9 mice per group). **(M)** Representative immunofluorescence images of transverse mouse ileum sections stained with anti-aSyn aggregate specific antibody. **(N)** Quantification of aSyn aggregate area as percentage of total area comprising the submucosa-muscularis region, presented as fold change versus untreated (n = 4-12 mice per group). **(O)** IL-17 levels (pg/mL) in plasma (n = 4-8 mice per group). **(P)** IL-1β levels (pg/mL) in SN (n = 4-10 mice per group). **(Q)** IL-17 levels (pg/mL) in SN (n = 4-8 mice per group). *p < 0.05, **p < 0.01, ***p < 0.001 by one-way ANOVA with Dunnet´s test (E, I, L, N and Q) or Kruskal-Wallis with Dunn´s test (A-C, F-G, J and O-P). Data are mean ± SEM. One-sample t-Test, comparing the mean values of PD1 or HC1 samples against the means of the other PD or HC groups was applied. The results indicated that we could not reject the null hypothesis (H_0), for panels A,B,E,F,G,I,L,P,Q as the mean differences were not statistically significant when compared to the reference values (Donors #1). Panel N was performed with only 1 sample animal/tissue from different HCs and PDs. In panels J and

O the differences between HC donors 2-4 and PD2-5 means are bigger them from the means of HC1 and PD1. Scale bars are 100 µm in D and 50 µm in the other images.

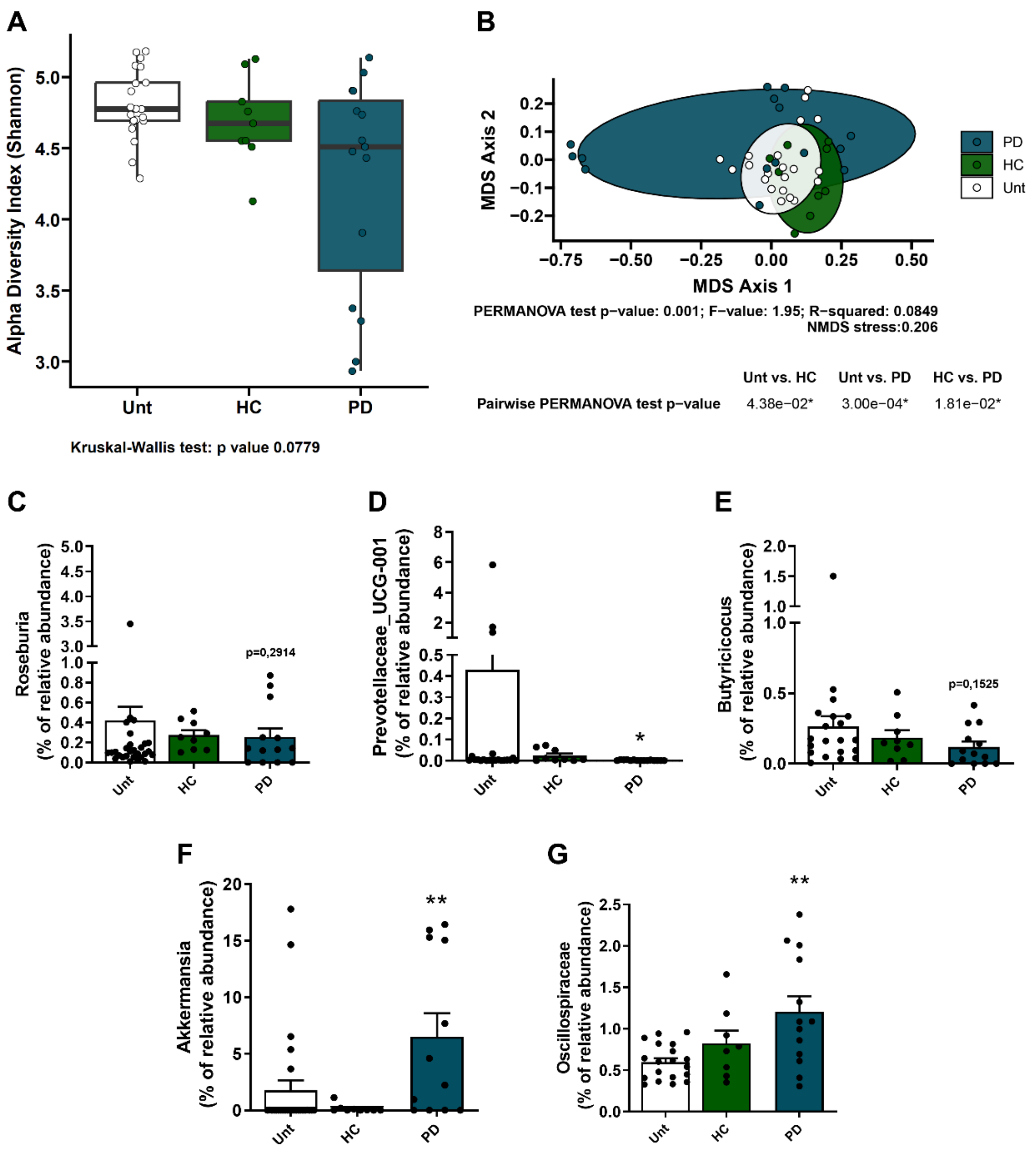

Supplementary Figure S4. Fecal microbiota diversity in transplanted mice.

(A) Alpha diversity measured using Shannon index at the OTU level in mice fecal samples (n= 21 Unt; 9 HC and. 15 PD). **(B)** Beta diversity was evaluated using Principal Coordinate Analysis (PCoA) based on the Bray-Curtis index of OTUs derived from mice fecal samples (n= 21 Unt; 9 HC and 15 PD). The PERMANOVA test showed significant differences between Unt and HC (*p=0.0432), Unt and PD (***p=0.0003), and HC and

PD (*p=0.0189). **(C-H)** The relative abundance of selected genera in mice fecal samples using statistical analysis methods such as PERMANOVA with DESeq2 Wald test or Kruskal-Wallis test. **(C)** *Akkermansia* (*padj HC vs. PD = 0.01); **(D)** *Roseburia* (p Unt vs. PD = 0,2914); **(E)** Prevotellaceae_UCG-001 (**padj Unt vs. PD = 0.00177); **(F)** *Butyricicocus* (p Unt vs. PD = 0.1525); **(G)** Oscillospiraceae (p Unt vs. PD = 0.3375); **(H)** Muribaculaceae_ge. The data are represented as mean ± SEM. A significance level of $*p \leq 0.05$ was used. (Information related to Figure 1)

Supplementary Table S3. Demographics and clinical information of selected PD patients and healthy control donors of terminal ileum biopsies

	Age	Clinical diagnosis	UPDRS	HOHEN & YAHR	MMSE	Constipation duration (Years)
**PD2**	72	2016	14	2	NCI	8
**PD3**	67	2007	10	2	NCI	16
**PD4**	80	2013	38	3	NCI	10
**PD5**	77	2011	32	2.5	NCI	10
**HC5**	72	n/a	n/a	n/a	NCI	none
**HC6**	73	n/a	n/a	n/a	NCI	none
**HC7**	71	n/a	n/a	n/a	NCI	none
**HC8**	72	n/a	n/a	n/a	NCI	none
**HC9**	84	n/a	n/a	n/a	NCI	none

UPDRS = Unified Parkinson's Disease Rating Scale (0-147); HY stage = Hoehn and Yahr scale (2: Bilateral disease, without impairment of balance). MMSE = Mini–Mental State Examination (24-30: NCI, no cognitive impairment). (Information related to Figures 3 and 4)

Supplementary Table S4. Relative abundance of relevant bacterial genera in the mucosa-associated microbiota of the terminal ileum in selected PD patients and healthy control donors.

Bacterial genus	HC6	HC7	PD3	PD5
Faecalibacterium	25.5	11.8	25.9	31.2
Prevotella	2.1	19.7	6	ND
Blautia	2.7	1.71	3	2
Roseburia	4.6	ND	2	2.5
Streptococcus	0.9	1.7	4.8	2.2
Alistipes	ND	ND	ND	1.6
Akkermansia	ND	0.5	ND	ND
Lactobacillus	ND	ND	ND	1.4
Bifidobacterium	ND	1.4	ND	ND

ND: not detected or below 0.1% relative abundance. (Information related to Figures 3 and 4)

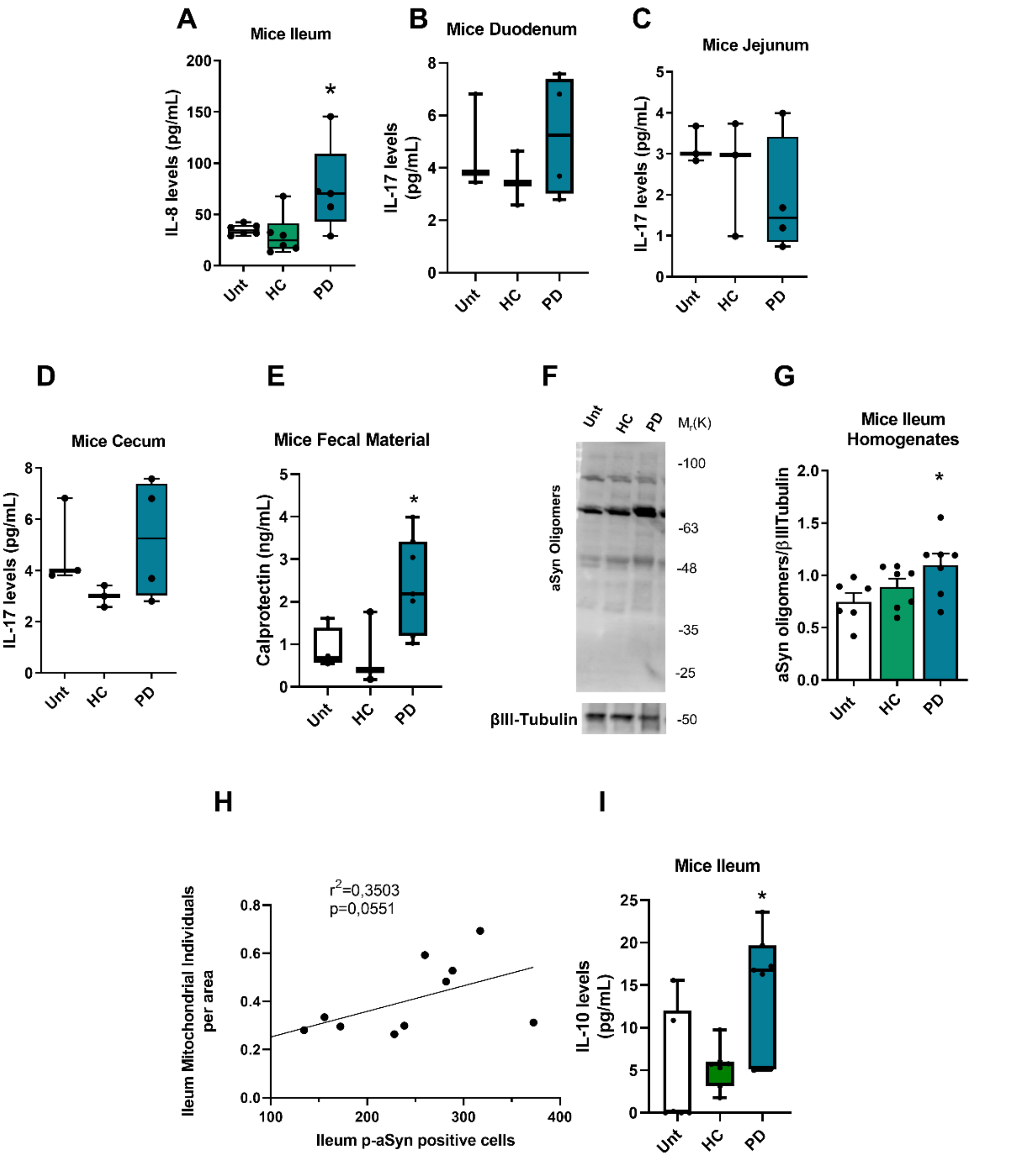

Supplementary Figure S5. Gut inflammatory markers.

Measurement of specific inflammatory cytokines. **(A)** Ileal IL-8 (n = 5-6 mice per group), **(B)** Duodenal IL-17 (n = 3-4 mice per group). **(C)** Jejunal IL-17 (n = 3-4 mice per group). **(D)** Cecal IL-17 (n = 3-4 mice per group). **(E)** Fecal material calprotectin levels (n = 3-7 mice per group). **(F)** Representative immunoblot of aSyn oligomers in ileum homogenates. The blots were re-probed for βIII-Tubulin to confirm equal protein loading. **(G)** Densitometric analyses of the aSyn levels normalized against βIII-Tubulin. The data are expressed as the ratio between aSyn and βIII-tubulin densitometry (n = 6-7 mice per group). **(H)** Correlation between mitochondrial fragmentation in the ileum and the increase of p-aSyn in the same area (n = 10, p= 0.0551, r^2 = 0.3551). **(I)** Ileal IL-10 (n =

6-7 mice per group). The statistical analysis showed a significance level of *$p < 0.05$ using one-way ANOVA with Dunnet´s test. The data represents the mean ± SEM. (Information related to Figure 3 and 4)

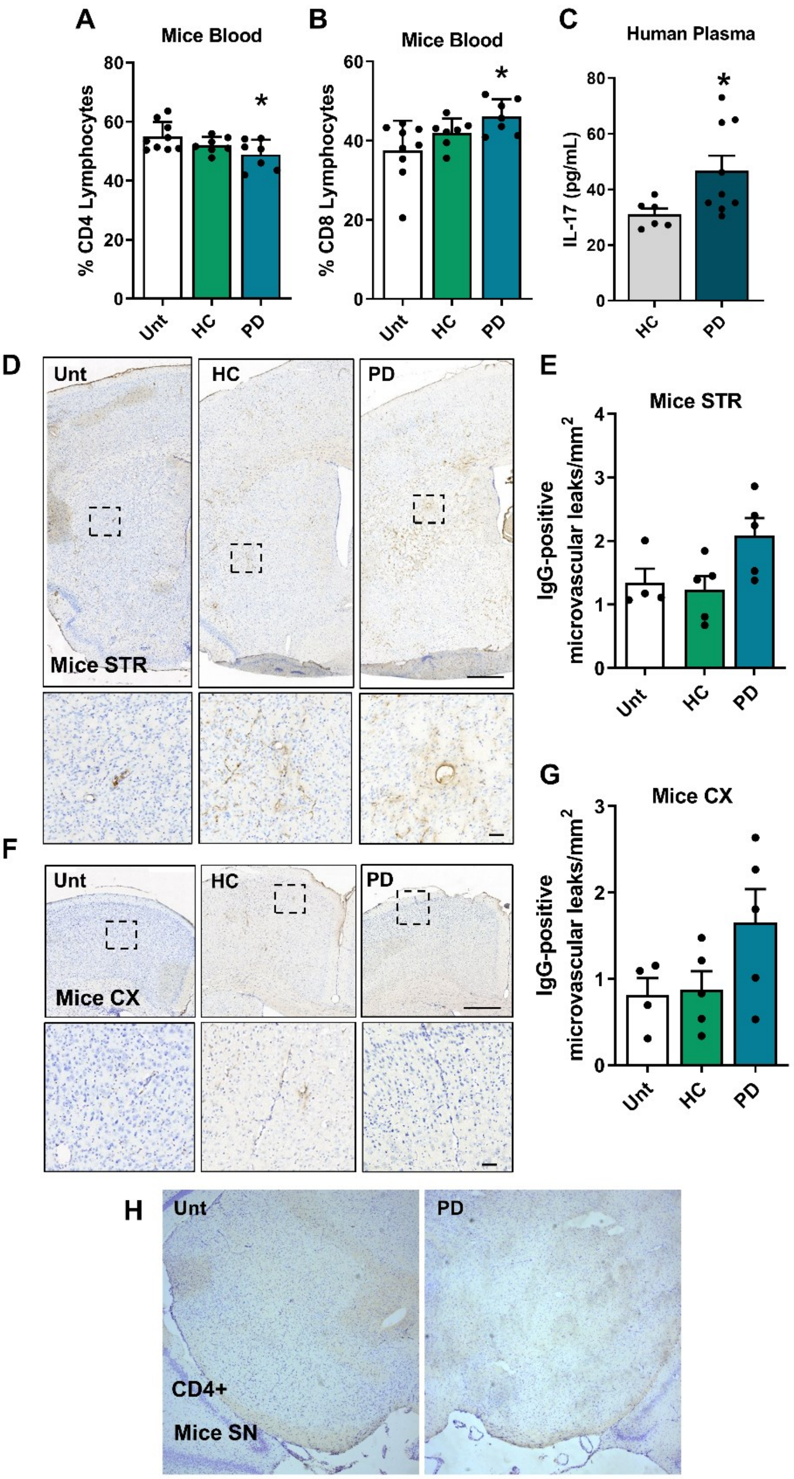

Supplementary Figure S6. Systemic inflammation and blood-brain barrier permeabilization (A) Percentage of CD4 lymphocytes in $CD45^{+}/CD3^{+}$ cell population

in the plasma as determined by flow cytometry (n = 7-9 mice per group). **(B)** Percentage of CD8 lymphocytes in $CD45^{+/}CD3^{+}$ cell population in the plasma as determined by flow cytometry (n = 7-9 mice per group). **(D-G)** Assessment of IgG-positive microvascular leaks in the STR and CX. **(D)** Quantification of IgG-positive microvascular leaks per mm^2 in STR (n= 4-5 mice per group). **(F)** Quantification of IgG-positive microvascular leaks per mm^2 in CX (n= 4-5 mice per group). **(H)** Assessment of CD4+ cells in the SN. The statistical analysis showed a significance level of *$p < 0.05$ using one-way ANOVA with Dunnet´s test and unpaired Student´s t test (C). Data are represented as mean ± SEM. Scale bars are 50 µm except in the upper box = 500 µm (Information related to Figure 5)

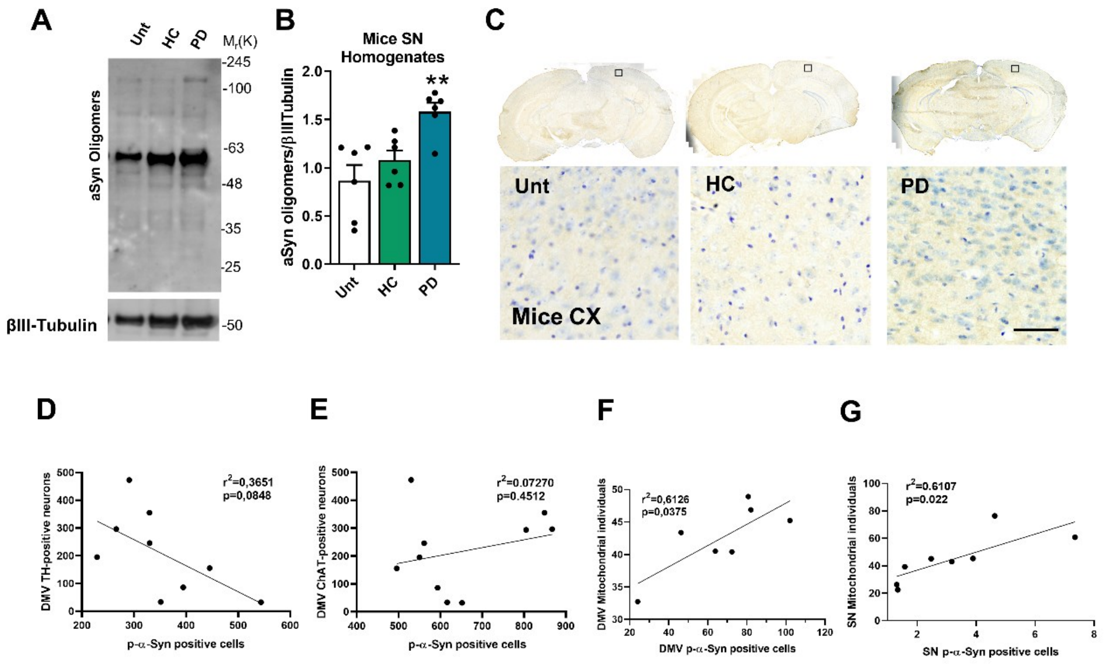

Supplementary Figure S7. Correlation between TH+ neurons, mitochondrial fragmentation, and aSyn in the DMV of HC and PD mice.

A) Representative immunoblot showing aSyn oligomers in mesencephalic homogenates. To confirm equal protein loading, the blots were re-probed for βIII-Tubulin. **(B)** Densitometric analyses of the levels of aSyn normalized against βIII-Tubulin. The data are expressed as the ratio between aSyn and βIII-tubulin densitometry (n = 6 mice per group). **(C)** Photomicrographs represent histology for aSyn aggregates immunoreactivity in the cortex from fecal material-treated mice. **(D-E)** Pearson correlation between DMV neurons and p-aSyn OD. (**D**) Correlation between the loss of TH-positive neurons in the

DMV with the increase of p-aSyn in DMV (n = 9, p= 0.0848, r^2= 0.3651); (**E**) Correlation between the loss of ChAT-positive neurons in DMV with the increase of p-aSyn in the DMV (n = 10, p= 0.4512, r^2= 0.0727). **(F-G)** Pearson correlation between mitochondrial individuals (fragmentation) and p-aSyn OD. (**F**) Correlation between mitochondrial fragmentation in the DMV with the increase of p-aSyn (n = 7, p= 0.0375, r^2= 0.6126). (**G**) Correlation between mitochondrial fragmentation in SN with the increase of p-aSyn (n = 8, p= 0.022, r^2= 0.6107). The statistical analysis showed a significance level of **p < 0.01 using ANOVA one-way with Dunnet´s test. Data are represented as mean ± SEM. Scale bar is 500 µm. (Information related to Figure 6 -8)

Supplementary Table S5. Demographics and clinical information of selected PD patients and controls *post-mortem* SN donors

	**Age**	**Disease duration**	**Braak stage**	**Diagnosis**
**PD**	81	25	5	Parkinson's disease
**PD**	81	29	5-6	Parkinson's disease
**PD**	77	11	5	Parkinson's disease
**PD**	77	11	4-5	Parkinson's disease
**PD**	74	19	5	Parkinson's disease
**HC**	83	n/a	n/a	Mild Argyrophilic grain disease I
**HC**	56	n/a	n/a	Small-cell lung cancer
**HC3**	73	n/a	n/a	Vascular encephalopathy, Hepatitis B virus
**HC4**	76	n/a	n/a	Mild Argyrophilic grain disease I

Information related to Supplementary Figure 7.

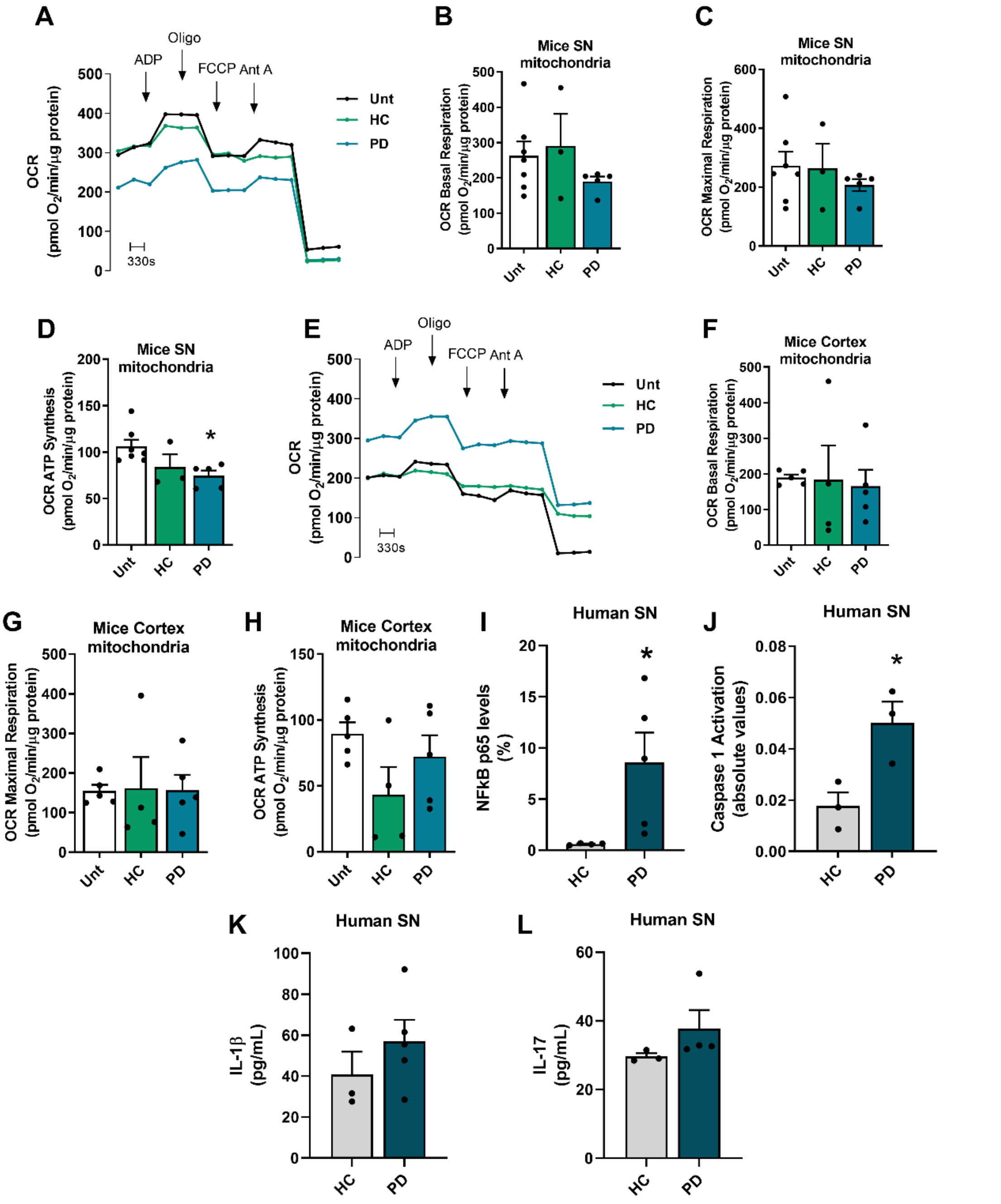

Supplementary Figure S8. Mitochondrial function and inflammasome activation. Isolated mesencephalic mitochondria were examined. **(A)** Representative graph displaying OCR. **(B)** Basal respiration; **(C)** Maximal respiration and **(D)** ATP synthesis. Values are pmol O_2/min/µg protein. (n= 3-7 mice per group). Isolated cortical mitochondria were examined. **(E)** Representative graph showing OCR in the cortex; **(F)** Basal Respiration; **(G)** Maximal respiration; **(H)** ATP synthesis. Values are pmol O_2/min/µg protein. **(E-H)** (n = 4-5 mice per group)**. (I-L)** Measurement of specific inflammatory cytokines in human SN. **(I)** NFκB. **(J)** Caspase 1. **(K)** IL-1β. **(L)** IL-17.

The statistical analysis showed a significance level of $*p < 0.05$ using one-way ANOVA with Dunnet´s test (C, F and H) or Kruskal-Wallis with Dunn´s test (B, D and G), and Mann-Whitney´s (L) or unpaired Student´s t-test (J). Nested t-test was performed in I and K due to limited number of human samples. Data represents mean ± SEM. (Information related to Figure 8**)**

Representative Western blot membranes

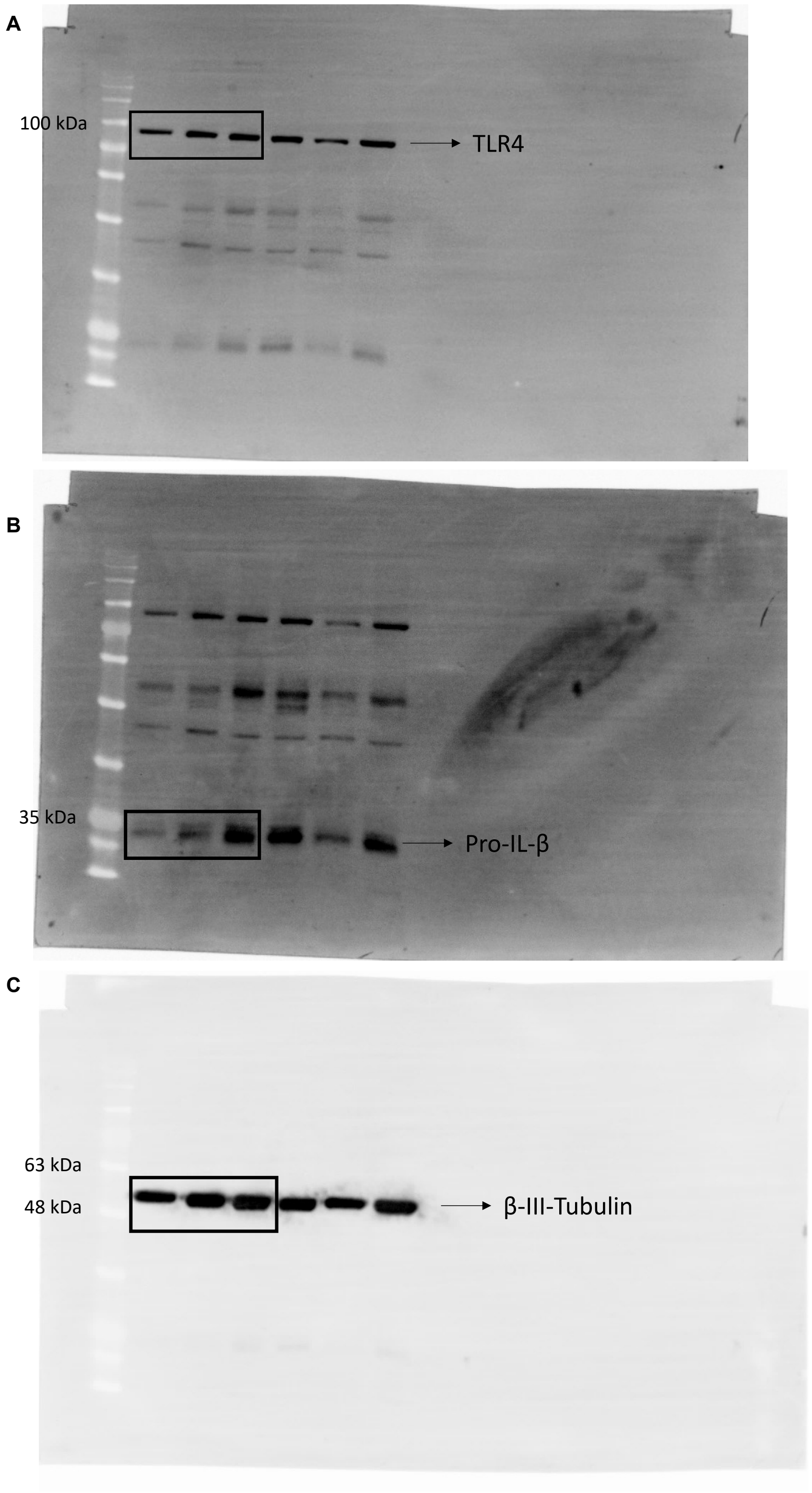

Figure 1. Whole representative Western blots of Figure 8C showing TLR4 at 95 kDa (A). ProIL-1β at 50 kDa (B) and βIII-Tubulin at 50 kDa (C).

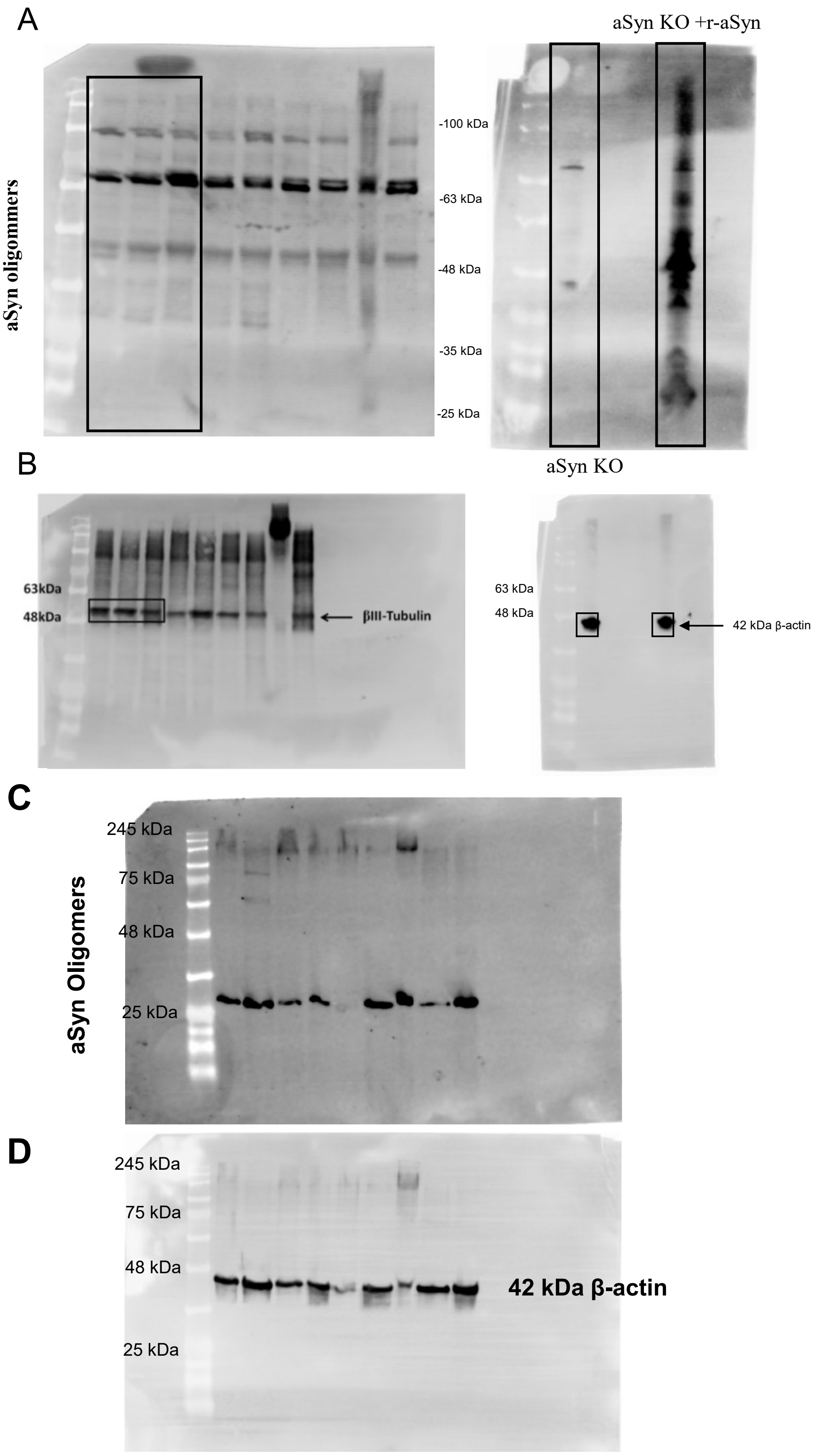

Figure 2. Whole representative Western blots of Supplementary Figure 5F showing Ileum aSyn oligomers in *WT* mice and in aSyn *KO* mice untreated and with recombinant aSyn (r-aSyn) (**A,C**) and βIII-Tubulin at 50 kDa or β-actin at 42 kDa (**B,D**).

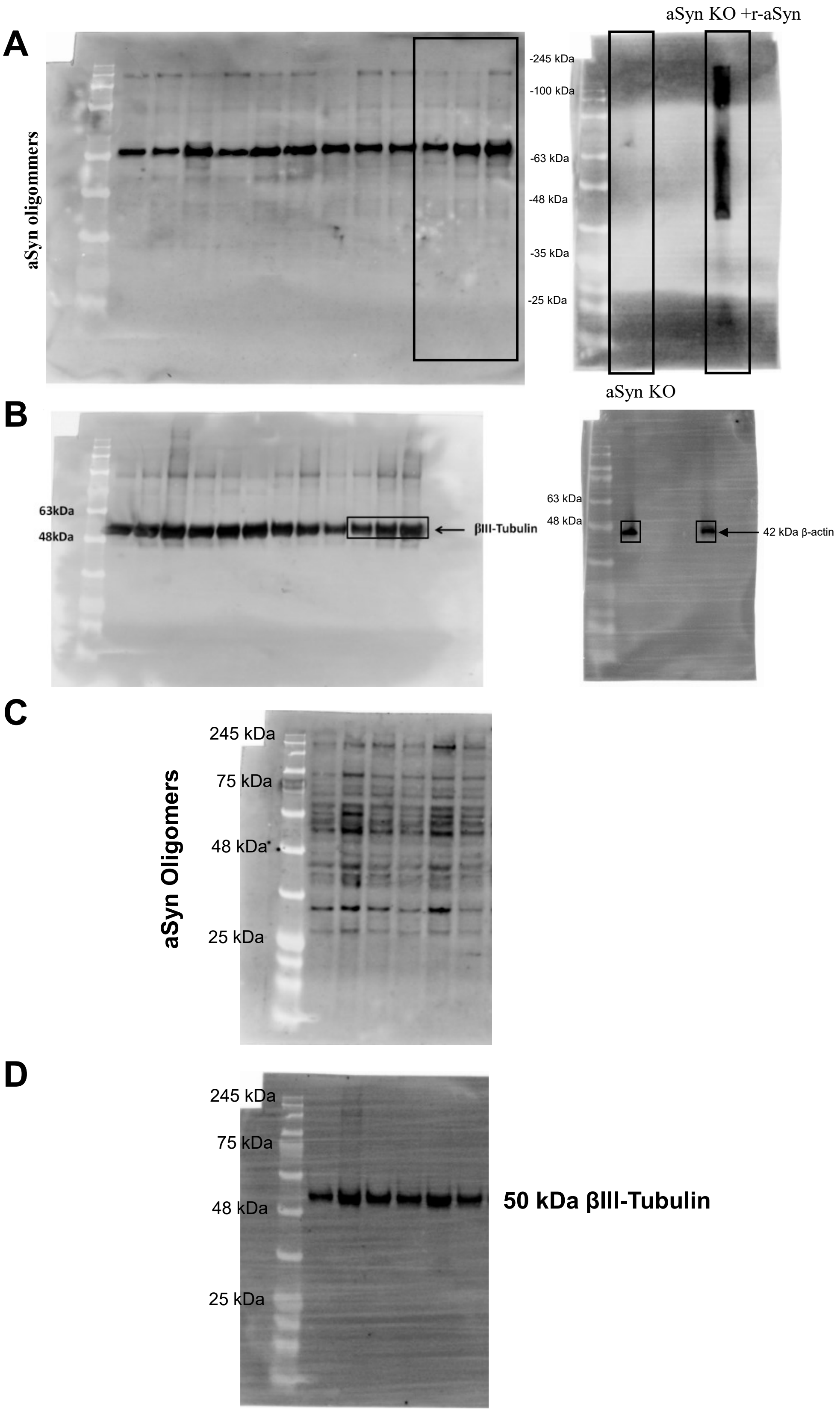

Figure 3. Whole representative Western blots of Supplementary Figure 7A showing aSyn oligomers in the SN of *WT* mice and untreated *KO* mice, and recombinant aSyn (r-aSyn) (**A,C**) and βIII-Tubulin at 50 kDa (**B,D**).
